# A new machine learning-based prediction model for subtype diagnosis in primary aldosteronism

**DOI:** 10.3389/fendo.2022.1005934

**Published:** 2022-11-23

**Authors:** Shaomin Shi, Yuan Tian, Yong Ren, Qing’an Li, Luhong Li, Ming Yu, Jingzhong Wang, Ling Gao, Shaoyong Xu

**Affiliations:** ^1^ Department of Endocrinology, Xiangyang Central Hospital, Affiliated Hospital of Hubei University of Arts and Science, Xiangyang, Hubei, China; ^2^ Department of Cardiology, Xiangyang Central Hospital, Affiliated Hospital of Hubei University of Arts and Science, Xiangyang, Hubei, China; ^3^ Department of General Medicine, Xiangyang Central Hospital, Affiliated Hospital of Hubei University of Arts and Science, Xiangyang, Hubei, China; ^4^ Department of Interventional Medicine, Xiangyang Central Hospital, Affiliated Hospital of Hubei University of Arts and Science, Xiangyang, Hubei, China; ^5^ Center for Clinical Evidence-Based and Translational Medicine, Xiangyang Central Hospital, Affiliated Hospital of Hubei University of Arts and Science, Xiangyang, Hubei, China

**Keywords:** primary aldosteronism, subtype diagnosis, machine learning, captopril challenge test, saline infusion test

## Abstract

**Introduction:**

Unilateral primary aldosteronism (UPA) and bilateral primary aldosteronism (BPA) are the two subtypes of PA. Discriminating UPA from BPA is of great significance. Although adrenal venous sampling (AVS) is the gold standard for diagnosis, it has shortcomings. Thus, improved methods are needed.

**Methods:**

The original data were extracted from the public database “Dryad”. Ten parameters were included to develop prediction models for PA subtype diagnosis using machine learning technology. Moreover, the optimal model was chose and validated in an external dataset.

**Results:**

In the modeling dataset, 165 patients (71 UPA, 94 BPA) were included, while in the external dataset, 43 consecutive patients (20 UPA, 23 BPA) were included. The ten parameters utilized in the prediction model include age, sex, systolic and diastolic blood pressure, aldosterone to renin ratio (ARR), serum potassium, ARR after 50 mg captopril challenge test (CCT), primary aldosterone concentration (PAC) after saline infusion test (SIT), PAC reduction rate after SIT, and number of types of antihypertensive agents at diagnosis. The accuracy, sensitivity, specificity, F1 score, and AUC for the optimal model using the random forest classifier were 90.0%, 81.8%, 96.4%, 0.878, and 0.938, respectively, in the testing dataset and 81.4%, 90.0%, 73.9%, 0.818 and 0.887, respectively, in the validating external dataset. The most important variables contributing to the prediction model were PAC after SIT, ARR, and ARR after CCT.

**Discussion:**

We developed a machine learning-based predictive model for PA subtype diagnosis based on ten clinical parameters without CT imaging. In the future, artificial intelligence-based prediction models might become a robust prediction tool for PA subtype diagnosis, thereby, might reducing at least some of the requests for CT or AVS and assisting clinical decision-making.

## 1 Introduction

Primary aldosteronism (PA) accounts for approximately 4-10% of hypertension; approximately 30% of PA is a unilateral subtype that is associated with a higher risk of cardiovascular complications and can be cured by surgery, whereas the bilateral subtype is best treated with medication (1–4). Diagnosis of the specific subtype in PA is of great significance to support the use of targeted treatments, and clinical guidelines suggest that after screening and confirmatory testing, subtype diagnosis of PA should be made by imaging, such as computerized tomography (CT) or adrenal venous sampling (AVS) (5). However, CT is unreliable for the differentiation of UPA from BPA. It was reported that in BPA, CT scanning was able to determine the bilateral diagnosis in only 46.3% of the cases (6), and the overall consistency between imaging and AVS was 56.3% (7). Meanwhile, other available procedures to discriminate UPA from BPA, such as aldosterone to renin ratio (ARR) and posture testing, have limited effectiveness and are not used in the clinical management of PA (6). Currently, AVS is still typically employed as the gold standard for the discrimination between UPA and BPA (5). However, AVS cannot be widely used based on its high cost, inefficiency, technical difficulty, and limited diagnostic accuracy as confirmed by postadrenalectomy follow-up, along with the inconsistency among diagnostic centers regarding its decision criteria (4). Thus, over the past decade, some scoring models have been developed to differentiate UPA from BPA based on clinical and biochemical parameters alone or in conjunction with imaging, yet these previously developed models have limited accuracy and external validity. Thereafter, improved methods for subtype diagnosis are needed.

Meanwhile, supervised machine learning technology has been used widely and is gaining recognition in medical research since it can automatically formulate computational models by processing the variables with complex relationships, and therefore, frequently presents optimal results compared with traditional methods (8, 9). Due to the aforementioned concerns, we aimed to develop new supervised machine learning algorithms based on ten easily available parameters extracted from screening and confirmatory tests to predict the subtypes of PA. Given the cost, dependence on radioactivity, and unreliability of available PA typing tests, this model may be particularly useful as a complementary approach to reduce at least some of the need for CT or AVS and to assist clinical decision-making.

## 2 Materials and methods

### 2.1 Study design and population

This study includes two parts: modeling and validating. The population data for modeling were downloaded from the public database, “Dryad Digital Repository”, from “Aldosterone reduction rate after saline infusion may be a novel clinical prediction of determining subtypes of primary aldosteronism” (https://Datadryad.org). Validation data were obtained from XiangYang Central Hospital, Affiliated Hospital of Hubei University of Arts and Science, China. Ethics approval was obtained in the original study by the ethics committee of Chiba University Graduate School of Medicine (7), and the external validation was approved by the ethics committee of Xiangyang Central Hospital, an affiliated hospital of Hubei University of Arts and Science.

The Dryad database, funded by the National Science Foundation (U.S.), maintains high-quality research data with the aim of forming an academic exchange for the protection and reuse of research data in scientific publications. The raw data of the present study were offered publicly by Nagano et al. in 2020 (7). The original study initially included 209 PA patients who underwent AVS during a 6-year enrollment period, and 25 patients were excluded in both the original and the present study, who were either incorrectly classified or lacked a definite classification of PA in the postoperative follow-up. Since the plasma aldosterone concentration (PAC) reduction rate and PAC after saline infusion test (SIT) were reported to be valuable for subtype diagnosis in the original study, 19 patients without SIT were further excluded in the present study. Finally, 165 patients (71 UPA, 94 BPA) were included in our analysis.

The models were validated in an independent external PA cohort from XiangYang Central Hospital, an affiliated hospital of Hubei University of Arts and Science, China. Forty-three consecutive patients with PA who underwent AVS and SIT from 1 May 2021 to 30 April 2022 were included retrospectively based on the following criteria: (1) inclusion criteria: patients with PA and who had undergone successful AVS for subtype diagnosis. (n=54) (2) exclusion criteria: (a) patients without accessible data. (n=0) (b) patients who lacked a definite or correct classification of PA in the postoperative follow-up. (n=6) (c) Patients who did not undergo SIT. (n=5). Finally, 43 patients were included (details are shown in **Supplementary Figure 1**).

### 2.2 Modeling process

#### 2.2.1 Data extraction

Data extraction in the modeling process included age (year), sex, systolic blood pressure (SBP, mmHg), diastolic blood pressure (DBP, mmHg), plasma renin activity (PRA, ng/ml/h), PAC (pg/ml), ARR, serum potassium (K, mmol/L), urine aldosterone (μg/day), ARR after 50 mg CCT, PRA after the furosemide standing test, PAC after SIT, PAC reduction rate after SIT (%), and the number of types of antihypertensive agents at diagnosis. Blood pressure was measured three times consecutively after the patient sat for at least 15 minutes, and the average value was adopted. Blood samples were collected from the patient in the recumbent position after they rested for at least 30 minutes, and commercial radioimmunoassay kits (FUJIREBIO, Japan) were applied to these samples for the measurement of PAC and PRA. For the measurement of cortisol concentrations, the IMMULYZE (Siemens K.K.) assay was used. Serum potassium was measured by standard methods. Before and during patient examinations, calcium channel blockers or alpha-l blockers were administered, while patients with a severe condition were also treated with diuretics or other antihypertensive drugs. To make a definitive diagnosis of PA, patients underwent one or more of the following tests: saline infusion test, captopril challenge test, or furosemide upright test; notably, 91.8% of patients underwent more than two of these tests. AVS was performed with 0.25 mg of adrenocorticotropic hormone for stimulation. A selectivity index (SI, ratio of cortisol level in the adrenal vein to that in the inferior vena cava) above 5 was considered the threshold for successful adrenal vein cannulation. UPA was highly suspected when the lateral index (LI, the ratio of aldosterone-to-cortisol ratio on the dominant adrenal side to that on the opposite side) was above 3 after ACTH infusion and was confirmed in the postoperative follow-up (Laparoscopic total removal of the adrenal gland was performed), as described in the original study (7).

#### 2.2.2 Model development

**Figure 1:**
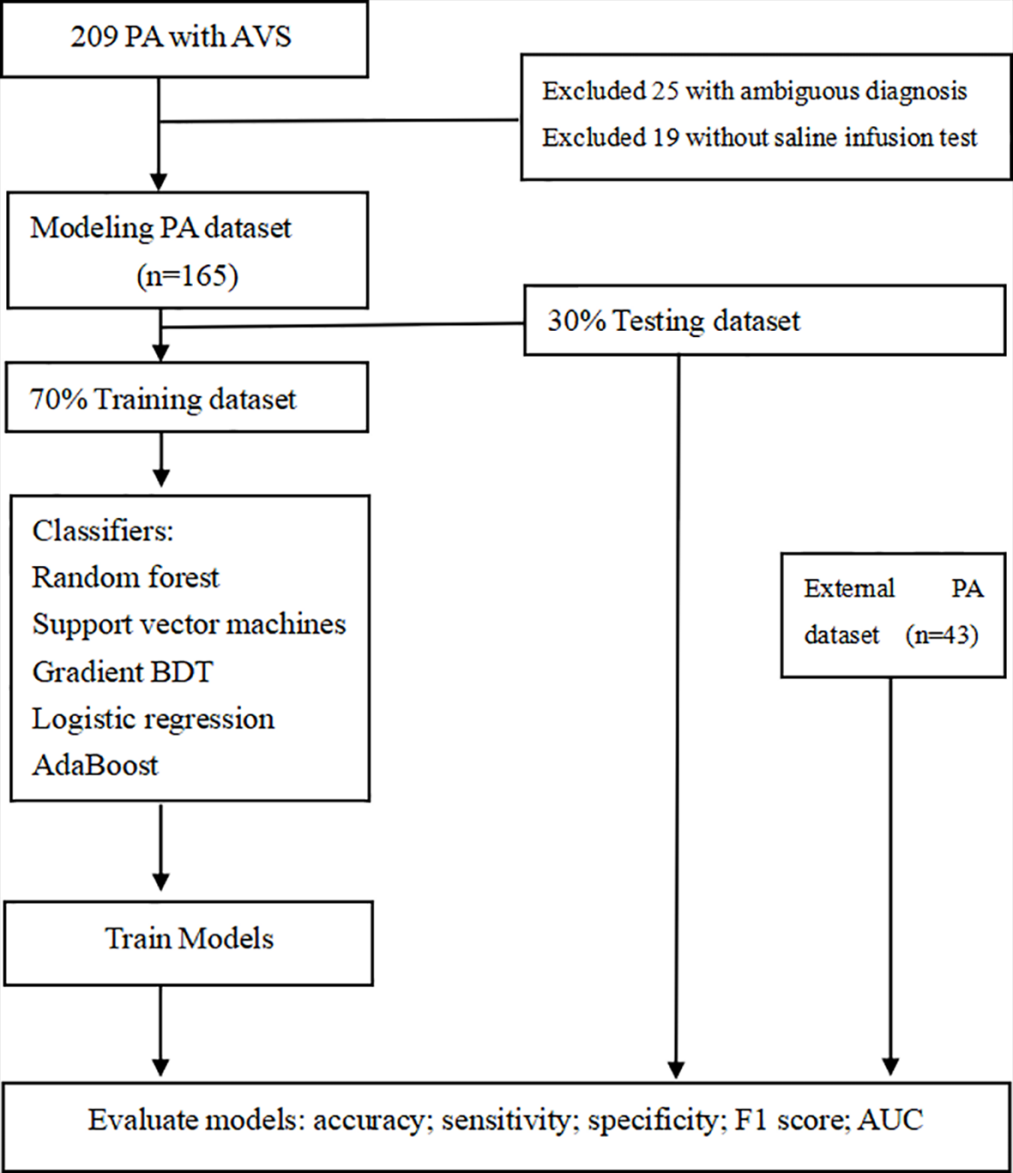
The workflow for developing machine learning-based models in this study.

Python 3.6.13 (library, scikit-learn) was used to develop and validate the machine learning-based algorithm. Patients with PA were stratified by subtype diagnosis and split randomly into either the training set (70%, N=115) or the testing set (30%, N=50) (random seed=1). To find an optimal prediction model, five supervised machine learning classifiers were used, including random forest (RF), support vector machines (SVM), gradient boosting decision tree (GBDT), logistic regression (LR), and AdaBoost. First, all possible available variables were used to build models, according to previous research reports (7, 10, 11). Two parameters, urinary PAC and PRA after the furosemide standing test, were removed due to their low importance for the prediction model, relatively poor availability, and absence from the external validation dataset. Furthermore, the accuracies of the training, testing, and external datasets were 100%, 88.0%, and 72.0%, respectively, which indicated that the model might be overfitting (as shown in **Supplementary Folder 1**). Thus, since PAC and PRA were highly correlated with ARR, it provided an additional justification for their removal, and subsequently, the model’s performance increased. Finally, prediction models were developed using the remaining ten parameters (age, sex, SBP, DBP, ARR, serum K, ARR after CCT, PAC after SIT, PAC reduction rate after SIT, number of types of antihypertensive agents at diagnosis). When models were trained with LR and SVM classifiers, the data were normalized by Z score standard transformation. Stratified tenfold cross-validation and grid search were used to search the optimal hyperparameters of classifiers to increase the performance of the models in the training cohort. In RF, Gini importance was used as a general measure of feature relevance. The accuracy, sensitivity, specificity, F1 score and area under the receiver operating characteristic curve (AUC) were calculated to evaluate the performance of the models. Predictive models were compared among classifiers, and the one with the best performance was selected; the optimal model was subsequently validated in an external PA dataset (as summarized in **Figure 1**).

### 2.3 Validation process

Data extracted for the validation process included age, sex, SBP, DBP, ARR, serum K, ARR after 50 mg CCT, PAC after SIT, PAC reduction rate after SIT (%), and number of types of antihypertensive agents at diagnosis. The measurements of PAC and PRA in the recumbent position were extracted and tested using commercial radioimmunoassay kits (Zhengzhou Antu Bioengineering Co., LTD, China), and cortisol concentrations were measured by the same method. The methods used to measure other variables were consistent with the original study (7). PA was diagnosed based on detection and confirmatory tests according to the guidelines of PA (5, 12). Saline infusion tests and captopril challenge tests were performed. AVS was performed without ACTH stimulation in the morning hours following overnight recumbency, which was inconsistent with the original study. However, it was not important since ACTH does not improve the diagnostic accuracy of AVS (5). An SI greater than 2 was considered the threshold for successful adrenal vein cannulation, and an LI greater than 2 was considered the cutoff for UPA, according to expert consensus (13).

### 2.4 Statistical analysis

Python 3.6.13 (library, scikit-learn) was used for development and validation of the models. STATA Version 16 (Stata Corporation, College Station, TX, USA) was used for statistical analysis. Continuous variables are presented as the mean ± standard deviation or the median with an interquartile range (25%,75%), and categorical variables are reported as counts with proportion (%). The student’s t test was used to analyze continuous variables when they were normally distributed, if not, the Mann-Whitney U test was used. The chi-square test was used for categorical variables. Accuracy was calculated as the primary endpoint to evaluate the performances of the prediction models, and secondary endpoints included sensitivity, specificity, F1 score, and AUC. Little missing data was replaced by the mean value (Details were shown in **Supplementary File 1**). A P value less than 0.05 (two-sided) was defined as significant.

## 3 Results

### 3.1 Basic characteristics

In the modeling dataset, 165 patients (71 UPA, 94 BPA) were included, while in the external dataset, we included 43 consecutive patients (20 UPA, 23 BPA) that underwent a successful AVS with accessible data. As expected, those with UPA had higher ARR, higher ARR after CCT, and higher PAC after SIT than those with BPA in both datasets (*P*<0.05). Accordingly, PAC reduction after SIT was significantly lower in the UPA subgroup than that in the BPA group (*P*<0.05). The serum potassium level was lower in patients with UPA than in those with BPA in both the modeling and the external dataset. Comparisons of other clinical and biochemical parameters were not significantly different between the two groups (as shown in **Table 1**). There were no differences for all evaluated characteristics between the training cohort and validation cohort (as shown in **Supplementary Table 1**). When comparing the entire modeling cohort to the external validation cohort, the patients in the external dataset were younger with higher DBP, lower PAC, and lower PAC reduction after SIT, and the patients took less antihypertensive agents at diagnosis, without any other significant difference.

**Table 1:** Basic characteristics of primary aldosterone patients in the modeling dataset and external dataset.

	Modeling dataset (165)				External dataset (43)				
	UPA (71)	BPA (94)	*P^1^*	Overall (165)	UPA (20)	BPA (23)	*P^1^*	Overall (43)	*P^2^*
Age (year)	54.5 ± 10.6	53.8 ± 11.4	0.701	54.1 ± 11.0	49.7 ± 10.8	48.7 ± 9.5	0.749	49.1 ± 10.0	0.008
Gender (Male%)	38 (53.5%)	41 (43.6%)	0.207	79 (47.9%)	9 (45%)	8 (34.8%)	0.494	17 (39.5%)	0.328
SBP (mmHg)	139 ± 18.5	141 ± 17.6	0.518	140 ± 18.0	142 ± 16.2	146 ± 15.3	0.451	144 ± 15.7	0.235
DBP (mmHg)	86 ± 12.0	86 ± 13.1	0.911	86 ± 12.6	92.5 ± 12.0	92.2 ± 12.7	0.941	92.3 ± 12.0	0.005
ARR (ng/ml/h)/(pg/ml)	1213 [660, 2630]	334 [206, 560]	<0.05	520 [300,1097]	623 [399, 1362]	205 [195,679]	0.012	429 [225, 816]	0.708
K(mmol/L)	3.2 ± 0.59	3.8 ± 0.3	<0.05	3.6 ± 0.52	3.25 ± 0.5	3.6 ± 0.3	0.010	3.4 ± 0.5	0.435
ARR after 50 mg captopril loading	1635 [555, 4490]	260 [144, 390]	<0.05	393 [209, 1390]	901 [320, 1595]	327 [255,719]	0.032	429 [255, 984]	0.280
PAC after SIT (pg/ml)	241 [95, 441]	50 [33, 82]	<0.05	78.6 [42.7, 225]	195 [155,272]	119 [102,154]	0.001	152 [112, 206]	<0.05
PAC reduction after SIT (%)	32.5 [-15.1, 59.9]	58.0 [46.9, 74.1]	<0.05	52.8 [27.9, 67.2]	1.75 [-6.6,30.4]	14.1 [1.7,24.3]	0.268	11.8 [-3.8.26.2]	<0.05
Antihypertensive agents at diagnosis	1.68 [1, 6]	1.26 [0, 4]	0.179	1.44 [0, 6]	1.70 [0, 3]	1.61 [0, 3]	0.963	1.70 [0, 3]	0.006

SBP indicates systolic blood pressure; DBP, diastolic blood pressure; ARR, aldosterone to renin ratio; K, serum potassium level; PAC, plasma aldosterone concentration; SIT, saline infusion test.

P^1^, UPA vs BPA; P^1^, Modeling dataset vs External dataset; “Antihypertensive agents at diagnosis” was presented as mean[min, max].

### 3.2 Evaluations of the developed models

**Figure 2:**
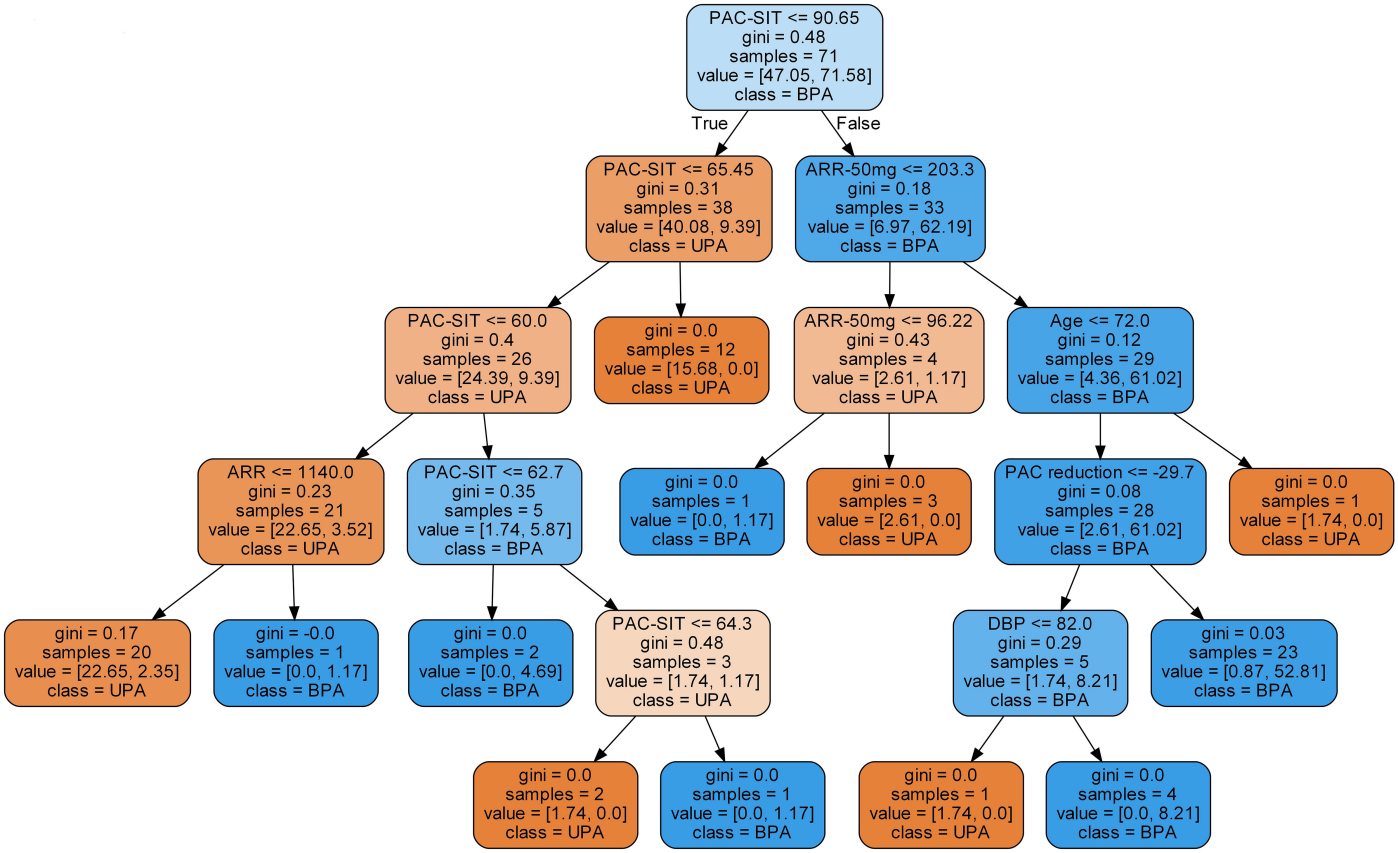
One of the prediction trees in the model using Random Forest classifier.

**Figure 3:**
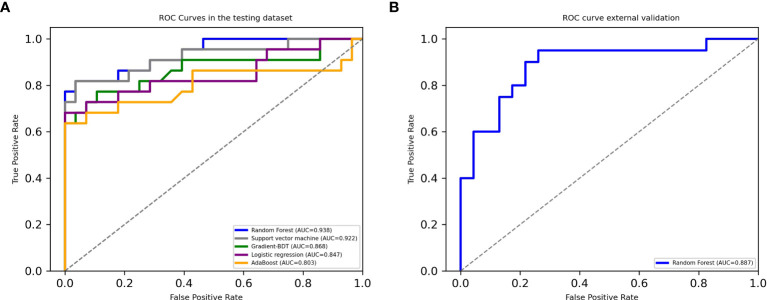
Receiver operating characteristic curves in the testing dataset **(A)** and in the external dataset using Random Forest classifier **(B)**.

**Figure 4:**
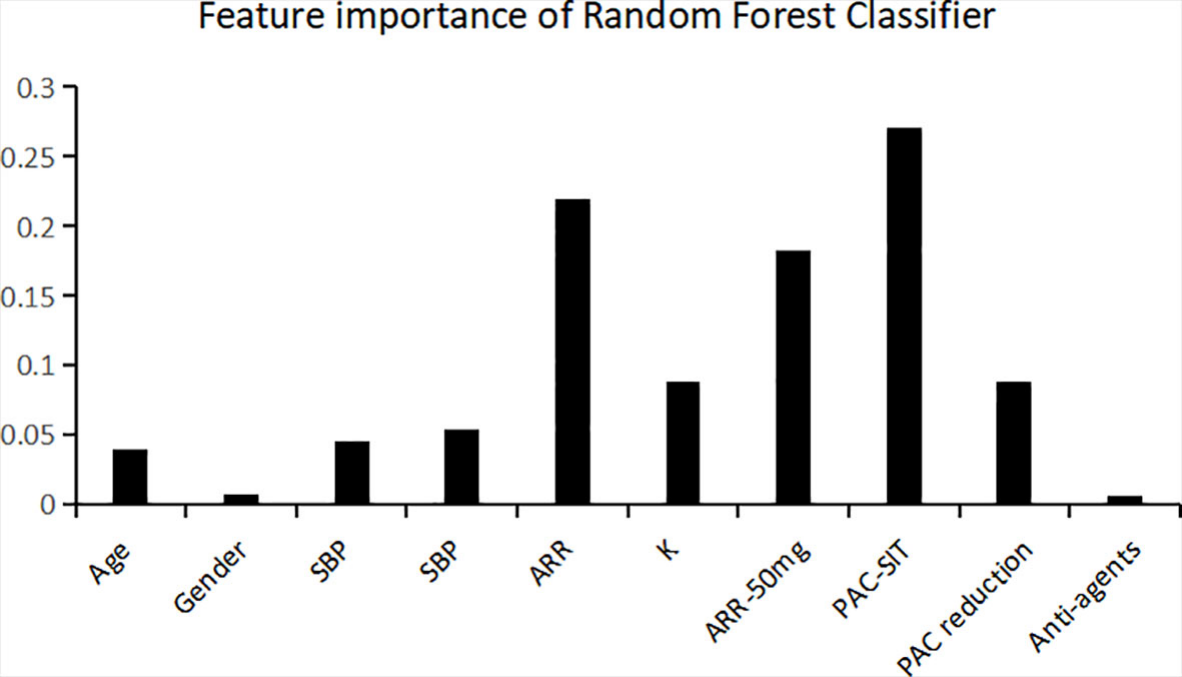
Relative variable importance for the accuracy of predicting unilateral primary aldosteronism using Random Forest classifier. SBP indicates systolic blood pressure; DBP, diastolic blood pressure; ARR, aldosterone to renin ratio; K, serum potassium level; PAC, plasma aldosterone concentration; SIT, saline infusion test; Anti-agents, the number of antihypertensive agents at diagnosis.

Among the classifiers assessed in the testing set, the best performance was observed in the model utilizing the RF classifier (as shown in **Table 2**). It comprised 42 classification trees with a maximum number of 12 splits. One of the classification trees from this model using the RF classifier is presented in **Figure 2**. The accuracy, sensitivity, specificity, F1 score, and AUC for the optimal model were 90.0%, 81.8%, 96.4%, 0.878, and 0.938, respectively (as shown in **Table 2** and **Figure 3a**). When validated in the external PA dataset, these values were 81.4%, 90.0%, 73.9%, 0.818 and 0.887, respectively (as shown in **Table 2** and **Figure 3b**; external validation with other classifiers shown in **Supplementary Figure 2**). The importance of each variable to the optimal prediction model was analyzed and is shown in **Figure 4** (details shown in **Supplementary Table 2**), with PAC after SIT, ARR, and ARR after CCT having greater importance. Moreover, the optimal model based on the RF classifier was developed as an online tool that allows access for clinical practice (https://github.com/shaominbaby/PA). A demonstration of how to apply the predictive model is shown in **Supplementary File 2** (Python code shown in **Supplementary Folder 2**), and a more friendly application is being developed (http://misetech.cn:83/xyszxyy.jsp, for Chinese users).

**Table 2:** Evaluations of the developed models using different classifiers in the testing dataset.

Classifiers	Accuracy	Sensitivity	Specificity	F1 score	AUC
Random Forest	0.900	0.818	0.964	0.878	0.938
Support Vector Machines	0.860	0.727	0.964	0.821	0.922
Gradient Boosting Decision Tree	0.820	0.591	1.000	0.743	0.868
Logistic Regression	0.860	0.682	1.000	0.811	0.847
Adaboost	0.820	0.682	0.929	0.769	0.803
RF-External validation	0.814	0.900	0.739	0.818	0.887

RF indicates Random Forest; AUC, the area under the receiver operating characteristic curve.

In addition, in order to apply the optimal model in clinical practice more accurately, further analysis was performed to integrate it with AVS to discriminate between UPA and BPA in the external dataset. When the subtype prediction probability was equal to or more than 0.8 (20.9%), the accuracy was 100%, while when it was equal to or more than 0.7, the accuracy was 95%. Thus if AVS was only performed in those whose subtype predicition probability < 0.7, then 51.2% of the AVS and 23.3% of the CT would have been avoided, with the sensitivity of 0.950 and specificity of 0.957 (As shown in **Supplementary Figures 3** and **4**).

### 3.3 Prediction model for PA subtype diagnosis without saline infusion test

Based on Chinese population, CCT and SIT were reported to be accurate alternatives for each other (14). Since CCT is much safer and more feasible compared with SIT (14), it is most commonly performed in the outpatient department. Thus, a predictive model for diagnosing the subtype of PA that does not rely on SIT was also developed using eight parameters (age, sex, SBP, DBP, ARR, serum K, ARR after CCT, and number of types of antihypertensive agents at diagnosis). It comprised 44 classification trees with a maximum number of 11 splits, using the RF classifier. The accuracy, sensitivity, specificity, F1 score, and AUC for internal validation were 84.0%, 72.7%, 92.9%, 0.800, and 0.904, respectively, while the external validation resulted in 69.8%, 60.0%, 78.2%, 0.649 and 0.780, respectively (details shown in **Supplementary Folder 3**). An additional online tool was developed to allow for this predictive model to be used in clinical practice (https://github.com/shaominbaby/PA-without-saline infusion test). The process of using this predictive model is shown in **Supplementary File 3**.

## 4 Discussion

### 4.1 The significance of the diagnosis of primary aldosteronism subtype

PA is associated with a higher risk of cardiovascular and cerebrovascular risk compared with essential hypertension (15). UPA and BPA are the two subtypes of PA. UPA can be cured by unilateral adrenalectomy, whereas the bilateral subtype is best treated with mineralocorticoid receptor antagonist (1–4). Establishing the subtype of PA during diagnosis timely and accurately is vastly important to optimize a specific treatment regimen and to prevent cardiovascular and cerebrovascular complications (13, 16).

### 4.2 Procedures for the diagnosis of primary aldosteronism subtype

Over the last few decades, many methods for the diagnosis of PA subtypes have been proposed. Although CT and magnetic resonance imaging (MRI) are the easiest and most accessible diagnostic tools, they are not reliable in differentiating subtypes of PA, with a pooled sensitivity of 68% and pooled specificity of 57% (16). Other commonly available procedures, such as the ARR and posture stimulation tests, have limited effectiveness (4, 12, 17). AVS is considered the gold standard, however its use is limited by its poor availability, technological difficulty, and invasive nature (4). Additionally, hybrid steroids in peripheral blood samples were reported to be valuable for discriminating PA subtypes (4, 16). A measurement of urinary 18-hydroxycortisol > 510 µg/24 h was reported to have a specificity of 100%, and plasma 18-oxocortisol > 4.7 ng/dL reportedly had a specificity of 99%, but both were without validation (18, 19). One study in 2011 reported that a PAC greater than 37.9 ng/dL after ACTH stimulation can be used to predict the presence of UPA, with a sensitivity of 91.3% and a specificity of 80.6% (20). Meanwhile, innovative imaging techniques, such as 6β-131-iodomethyl-19-norcholesterol (NP-59) scanning and 11C-metomidate positron emission tomography (PET)-CT scanning, have been attempted, yet they currently lack adequate effectiveness (4, 5, 21). Over the past decade, conventional scoring systems were developed to discriminate UPA from BPA based on demographics, biochemical parameters or imaging, with a sensitivity ranging from 32-95% and specificity ranging from 46-100% (4, 22–26); however, these scoring systems either lacked external validation or demonstrated low-to-modest performance when validated (17), indicating that they lack generalizability and are not reliable (27).

### 4.3 Comparison with previous studies

Machine learning is based on nonlinear algorithms, and consequently, can compute multidimensional variables simultaneously resulting in consistent levels of increased accuracy. This tool has been gaining increasing attention and recognition in medical decision-making (8).

Currently, the SIT and CCT tests are most commonly used in clinical practice for subtyping PA (6). In the present study, we developed a new prediction model of UPA using machine learning technology based on 10 widely available demographic and biochemical parameters, which is the first to combine the results of both CCT and SIT with machine learning. Among the five classifiers that were developed, the model using the RF classifier exhibited the best performance in the testing dataset (optimized sensitivity of 81.8%, specificity of 96.4%, and AUC of 0.938), and the performance was not poor in the external validating dataset (optimized sensitivity of 90.0%, specificity of 73.9%, and AUC of 0.887). The most important variables contributing to our prediction model were PAC after SIT, ARR, and ARR after CCT; these observations are consistent with the original study, which showed that PAC after SIT may be a novel predictor for subtype diagnosis in PA (7).

Moreover, the present prediction model did not rely on adrenal imaging, which may be another advantage, since it will be very valuable at least when the adrenal imaging is ambiguous. CT is always the first step for subtyping PA, but it is not always reliable, with a limited sensitivity and specificity (16). and it is expensive and requires radioactivity. If a part of patients with BPA subtype especially those with a high probability can be determined during the essential screening and confirmation process by a developed model, then at least this subset may benefit by avoiding further examination. Accordingly, further analysis showed that in the external dataset, if AVS was only performed in those whose subtype predicition probability < 0.7, it would achieve the accuracy of 95%, sensitivity of 0.950 and specificity of 0.957, and 51.2% of the AVS and 23.3% of the CT would have been avoided. So, the easily available prediction model can be a complement to the diagnostic process of PA and may be optimized further in the future. Notably, at our institution, only patients with a high probability of UPA who may potentially need surgery are assessed *via* AVS. Thus, the proportion of UPA in our external dataset was 46.5%, which is higher than that of the modeling dataset and previous reports (6, 28).

In addition, the diagnostic procedure of PA is relatively complex and burdensome compared to other diseases. CCT is much safer and more feasible as an outpatient procedure compared with SIT (14). Thus, another prediction model was developed to enable more PA patients to be treated on an outpatient basis. It used 8 parameters, excluding SIT, with a sensitivity, specificity, and AUC of 72.7%, 92.9% and 0.904, respectively, for the internal validation and 60.0%, 78.2% and 0.780, respectively, for the external validation. Although this model was not as effective as the former model we developed, its importance lies in its ability to meet actual clinical needs in our center and it may continue to be optimized in the future. Accordingly, two online tools were developed.

Over the past two years, several different machine learning-based prediction models for subtyping PA with different characters have been developed (as shown in **Supplementary Table 3**). Our model outperformed the model from Buffolo et al., whose accuracy was 82.0% for internal validation and 75.3% for external validation (10). The performance of our model from internal validation is comparable to that of Eisenhofer et al.’s prediction model, which was based on the profiles of seven steroids. Although their external validation model showed much better performance, with a sensitivity of 100% and specificity of 98%, it should be noted that they aimed to diagnose UPA due to KCNJ5 variants. When applied to wild-type KCNJ5, its performance was poor, with an AUC of only 0.716, and the validation sample size was small, at only 10% of the entire sample (12). Moreover, in clinical practice, the measurement of steroids is not always available. Burrello et al. tailored a prediction model to identify UPA specifically for when AVS was unilaterally successful and contralateral suppression was present to avoid the repetition of AVS, with an accuracy of 84.6% and without external validation (11). Another machine learning prediction model was developed to discriminate UPA from BPA based on six parameters, combining 3 biochemical measurements and 3 CT-related parameters, but its performance did not excel. However, a flow-chart integrating the 20-point scoring system was also developed, with an accuracy of 96.3%, and it enabled almost half of AVS procedures to be avoided, with similar performance in the external validation (17). In addition, Kaneko et al. recently developed a model aimed at predicting PA subtype in general practice settings using 21 available clinical and routine biochemical variables (29).

As stated above, models developed across varying institutions were based on different datasets, using diverse variables, and with specific aims and respective criteria. Thus, it is difficult to compare among them. Further prospective studies from large, multicenter cohorts are needed to reassess the prediction models before they are routinely implemented in clinical practice.

In the present study, all the UPA patients underwent total unilateral adrenalectomy, but recently it was reported that with the improvement of skills, minimally invasive partial adrenalectomy presented a higher rate of complete clinical success and shorter length of hospital stay, compared with total adrenalectomy (30). In addition, robot-assisted partial adrenalectomy has been successfully and effectively used for the treatment of PA (31). In the rapidly developing era of big data and intelligence, new prediction models for subtyping PA integrated into diagnostic flow-charts may improve the accuracy further, and both the diagnosis and treatment of PA will be optimized gradually.

### 4.4 Strengths and limitations

Our prediction model was validated in both the testing dataset and external dataset based on easily available parameters. It did not rely on CT imaging, which might be valuable at least when the adrenal imaging is ambiguous, and reduce at least some of the requests for CT or AVS for specific patients especially those with a high probability of BPA subtype, and it combined the results of both CCT and SIT with machine learning firstly. In addition, a second prediction model was developed which may be particularly useful in the outpatient setting in our centre. However, some limitations must be addressed. First, this is a retrospective study, and it cannot be guaranteed that all cases of PA have been correctly classified. Thus, further prospective studies are necessary to validate our prediction model. Second, similar to previous models, our model failed to confirm aldosterone hypersecretion side. Thirdly, the sample sizes of the modeling and external datasets were not large. Finally, this study performed model development and external validation in patients with PA from Japan and China, respectively. Thus, further testing in geographically and racially diverse populations is needed.

## 5 Conclusion

The development of new procedures to classify curable UPA from BPA is important, yet challenging. Until now, the complexity of subtyping has limited the use of optimized treatment for patients with PA. Here, using machine learning technology we developed a new predictive model for PA subtype diagnosis based on ten clinical parameters independent of CT imaging, which might be valuable when the adrenal imaging is ambiguous and reduce at least some of the requests for CT or AVS. In addition, we improved a SIT-free model with relatively good predictive value, which would be most useful for streamlining procedures in an outpatient setting in our centre. In the future, artificial intelligence-based prediction models might continue to improve and become a robust prediction tool to assist clinical decision-making.

## Data availability statement

The original contributions presented in the study are included in the article/**Supplementary Material**. Further inquiries can be directed to the corresponding authors.

## Ethics statement

Ethics approval was obtained in the original study by the ethics committee of Chiba University Graduate School of Medicine, and the external validation was approved by the ethics committee of Xiangyang Central Hospital, an affiliated hospital of Hubei University of Arts and Science. Written informed consent for participation was not required for this study in accordance with the national legislation and the institutional requirements.

## Author contributions

SS, LG, and SX conceived and designed the study. SS, YT, YR, Q’AL, LL, MY, and JW contributed to the data extraction. SS performed the analysis, interpreted the results and wrote the first draft. SX contributed to the revision of the final report and took the responsibility for the integrity of the data analysis. All authors read and approved the final version of manuscript.

## Funding

The study was partly supported by the Young Talents Project of Hubei Provincial Health Commission, China (Grand number WJ2021Q012); Science and Technology Research Key Project of Education Department of Hubei Province, China (Grand number D20212602); Sanuo Diabetes Charity Foundation, China.

## Acknowledgments

This study uses data from Dryad database. We thank the authors of the original study (Nagano H and his colleagues, Graduate School of Medicine, Chiba University Chiba 260-8670, Japan) for their excellent work.

## Conflict of interest

The authors declare that the research was conducted in the absence of any commercial or financial relationships that could be construed as a potential conflict of interest.

## Publisher’s note

All claims expressed in this article are solely those of the authors and do not necessarily represent those of their affiliated organizations, or those of the publisher, the editors and the reviewers. Any product that may be evaluated in this article, or claim that may be made by its manufacturer, is not guaranteed or endorsed by the publisher.
