# Supplementary material for: A new machine learning-based prediction model for subtype diagnosis in primary aldosteronism
Source: Front Endocrinol (Lausanne). 2022 Nov 23;13:1005934. doi: 10.3389/fendo.2022.1005934 (PMC9728523; doi:10.3389/fendo.2022.1005934)

1. **Application process of prediction model**
2. Download and install “Python” software, then download and install “Anaconda”, and use “Anaconda” to configure “Jupyter” (one of the application environment of python).
3. Download the files from “https://github.com/shaominbaby/PA”, and put them in python’s default directory list.

3.Open and run the file “website - PA.ipynb” in Jupyter.


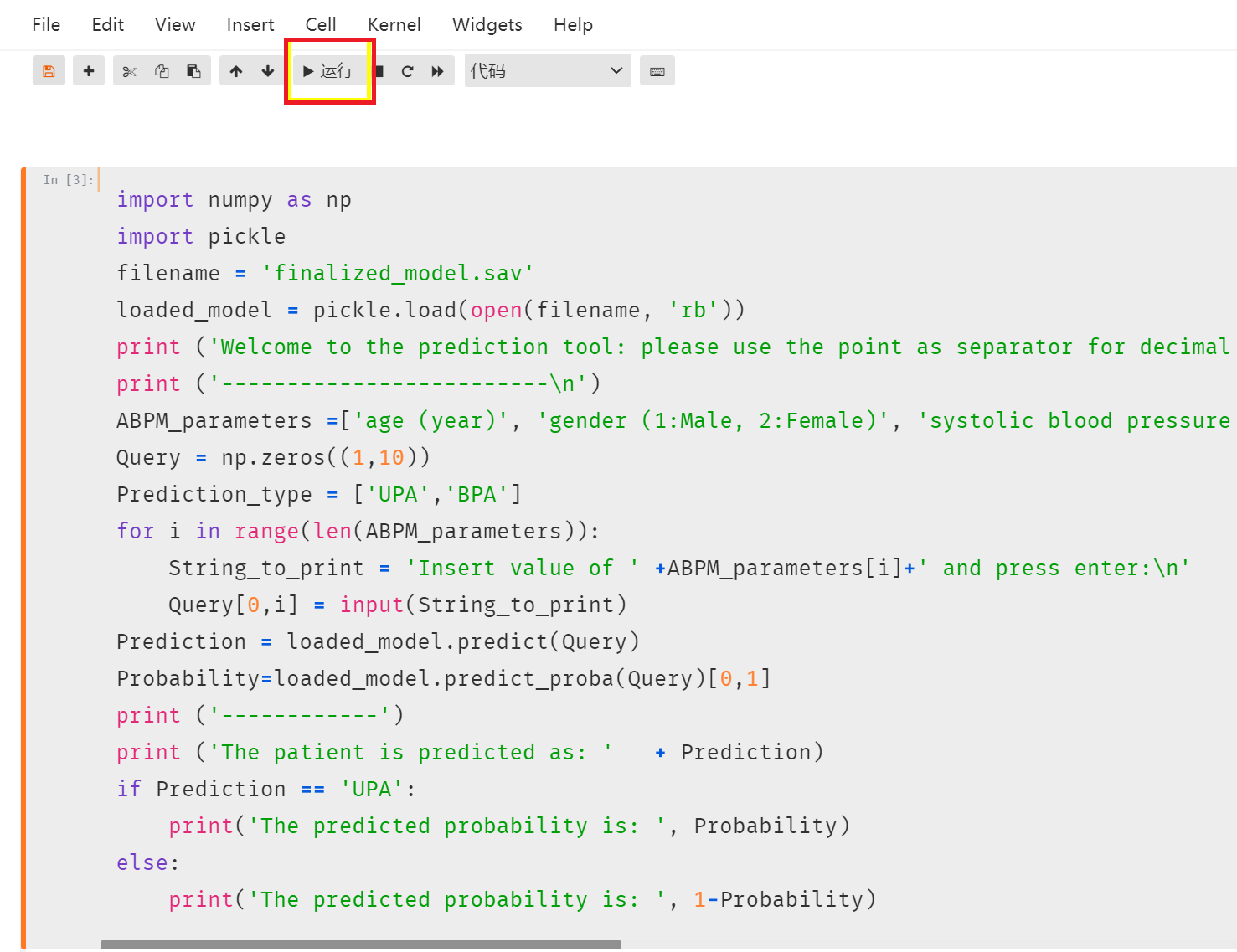


4.Enter the patient's age as promoted.


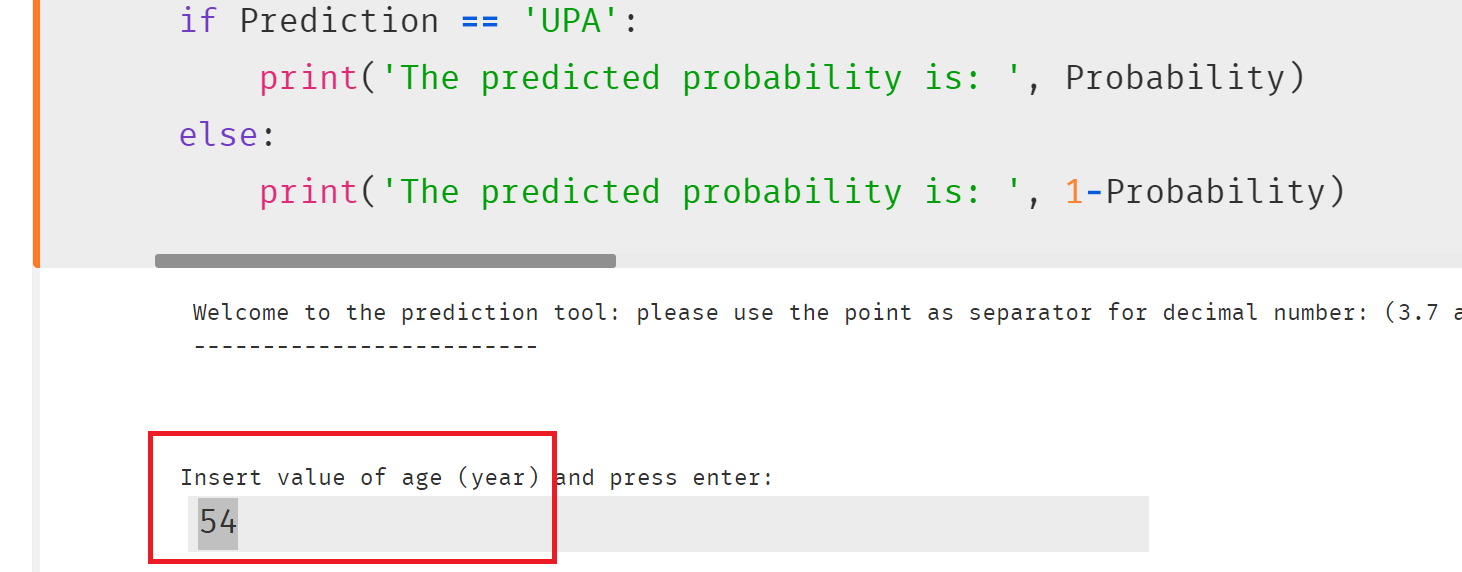


5.Enter the patient's other parameters (Gender, SBP, DBP, ARR, K, ARR after CCT, PAC after SIT, PAC reduction rate after SIT, types of antihypertensive agents) as promoted.

**
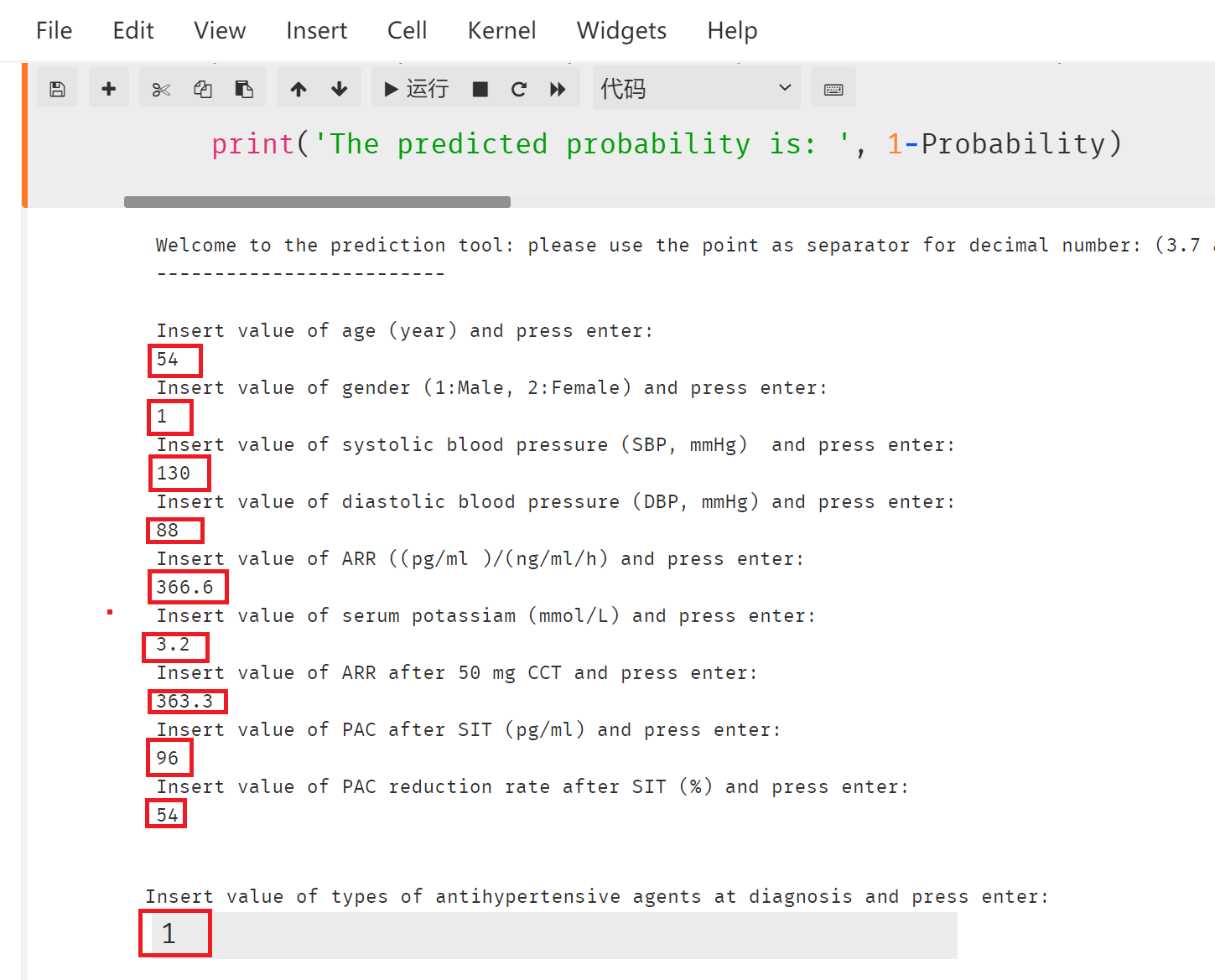
**

6.Press enter, then the predicted subtype of PA and probability will be displayed automatically.

**
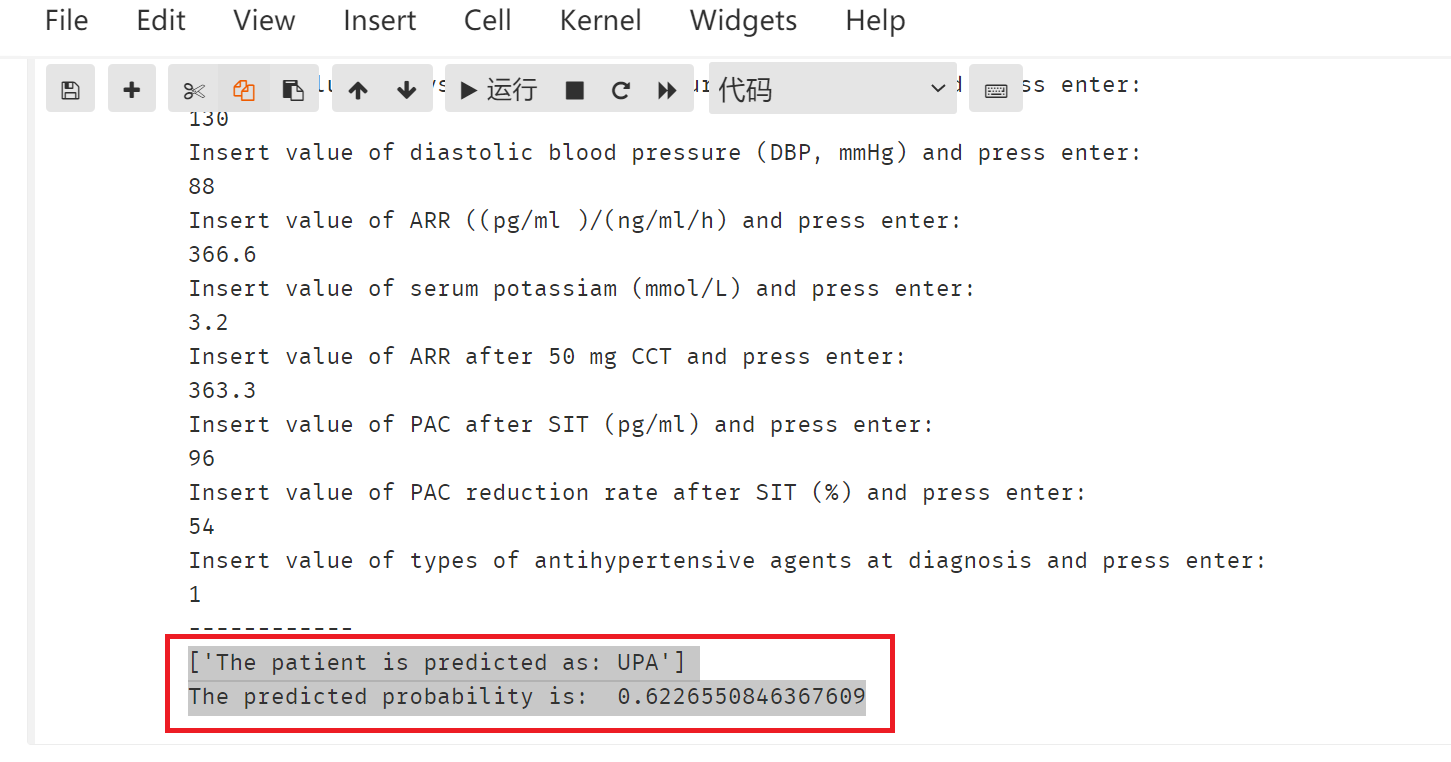
**

1. **Application process of prediction model without saline infusion test**

1.Download and install “Python” software, then download and install “Anaconda”, and use “Anaconda” to configure “Jupyter” (one of the application environment of python).

2.Download the files from “https://github.com/shaominbaby/PA without saline infusion test”, and put them in python’s default directory list.

3.Open and run the file “website - PA without SIT.ipynb” in Jupyter.


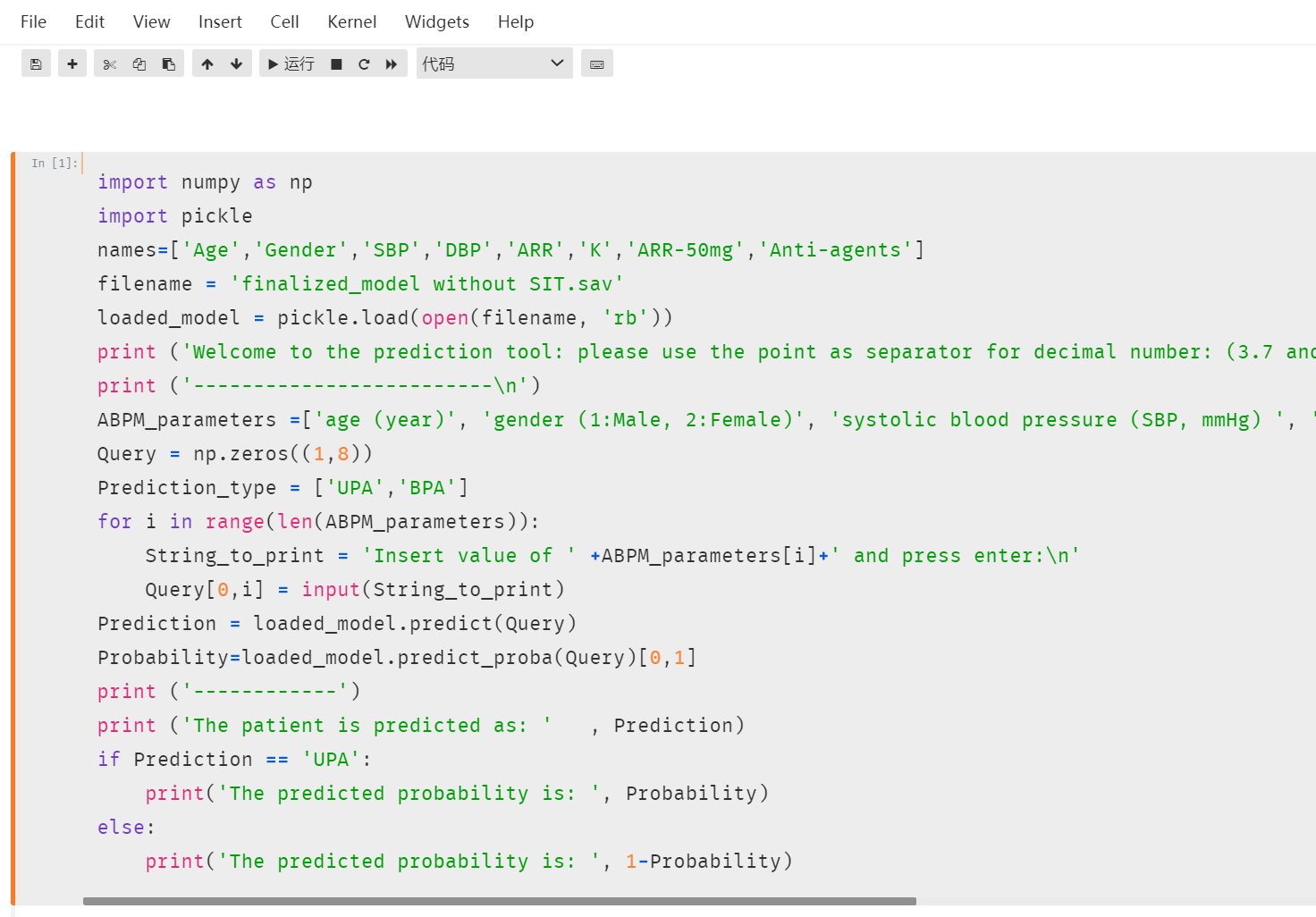


4.Enter the patient's age as promoted.


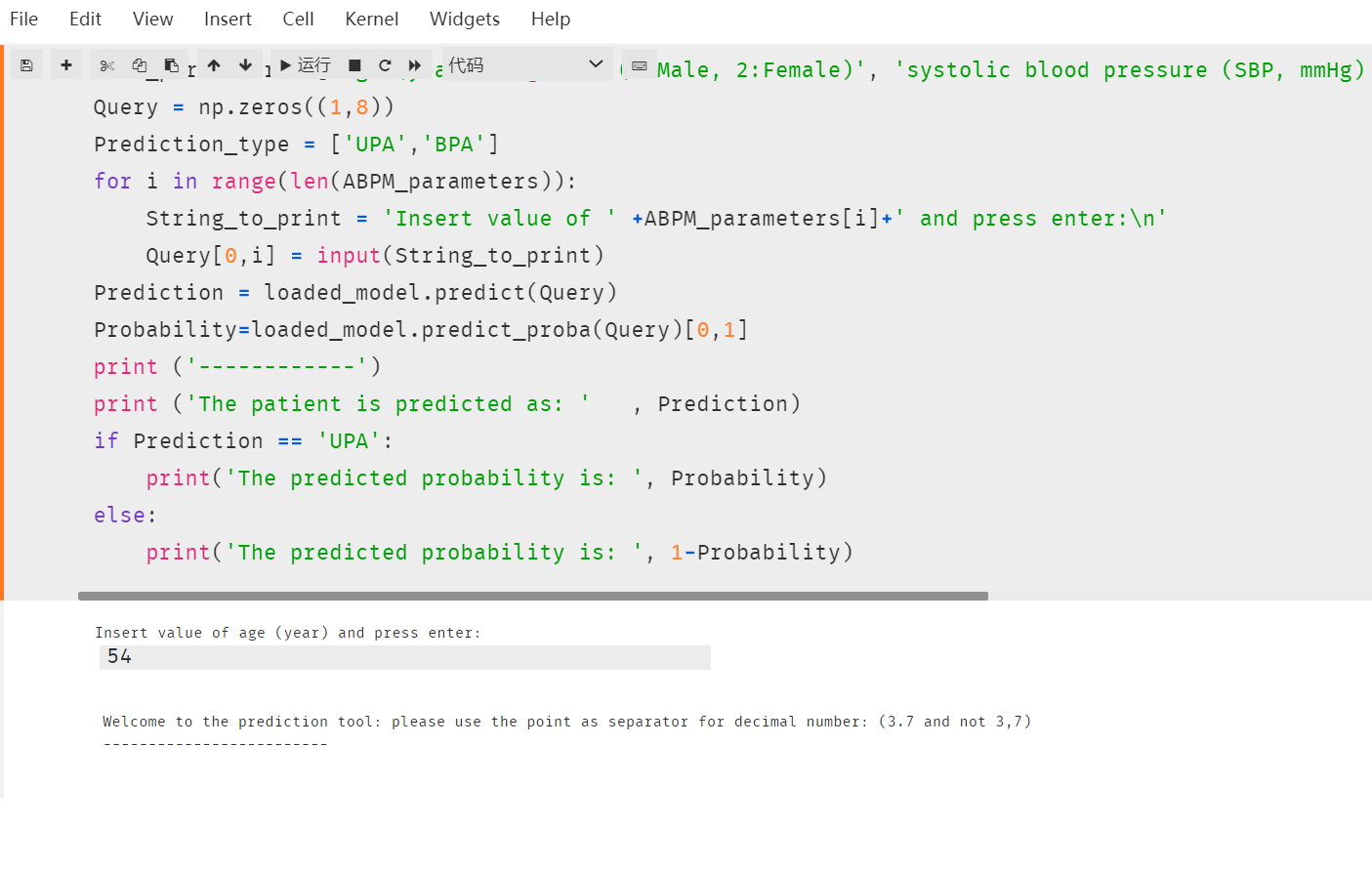


5.Enter the patient's other parameters (Gender, SBP, DBP,ARR,K,ARR after CCT, types of antihypertensive agents) as promoted.


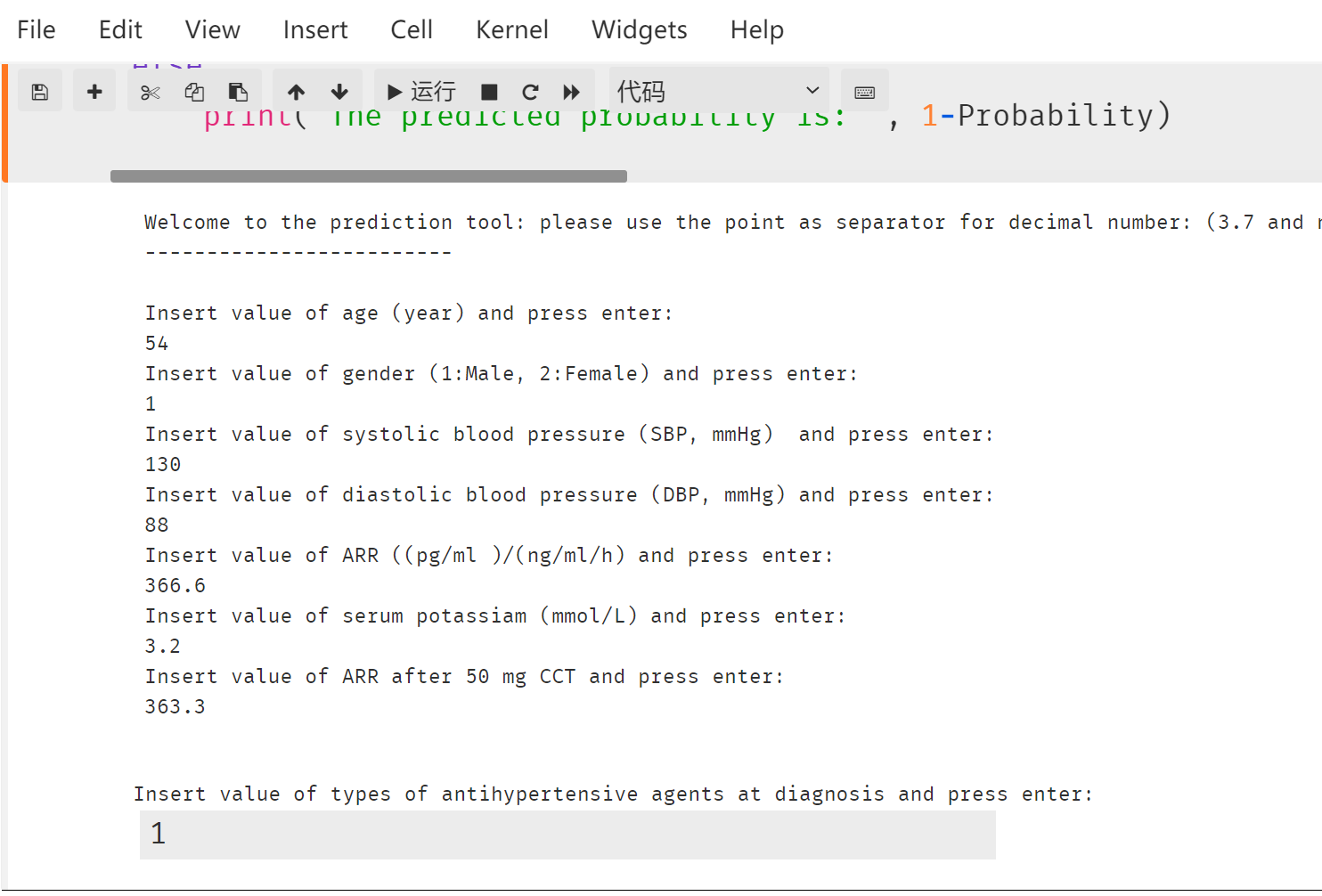


6.Press enter, then the predicted subtype of PA and probability will be displayed automatically.


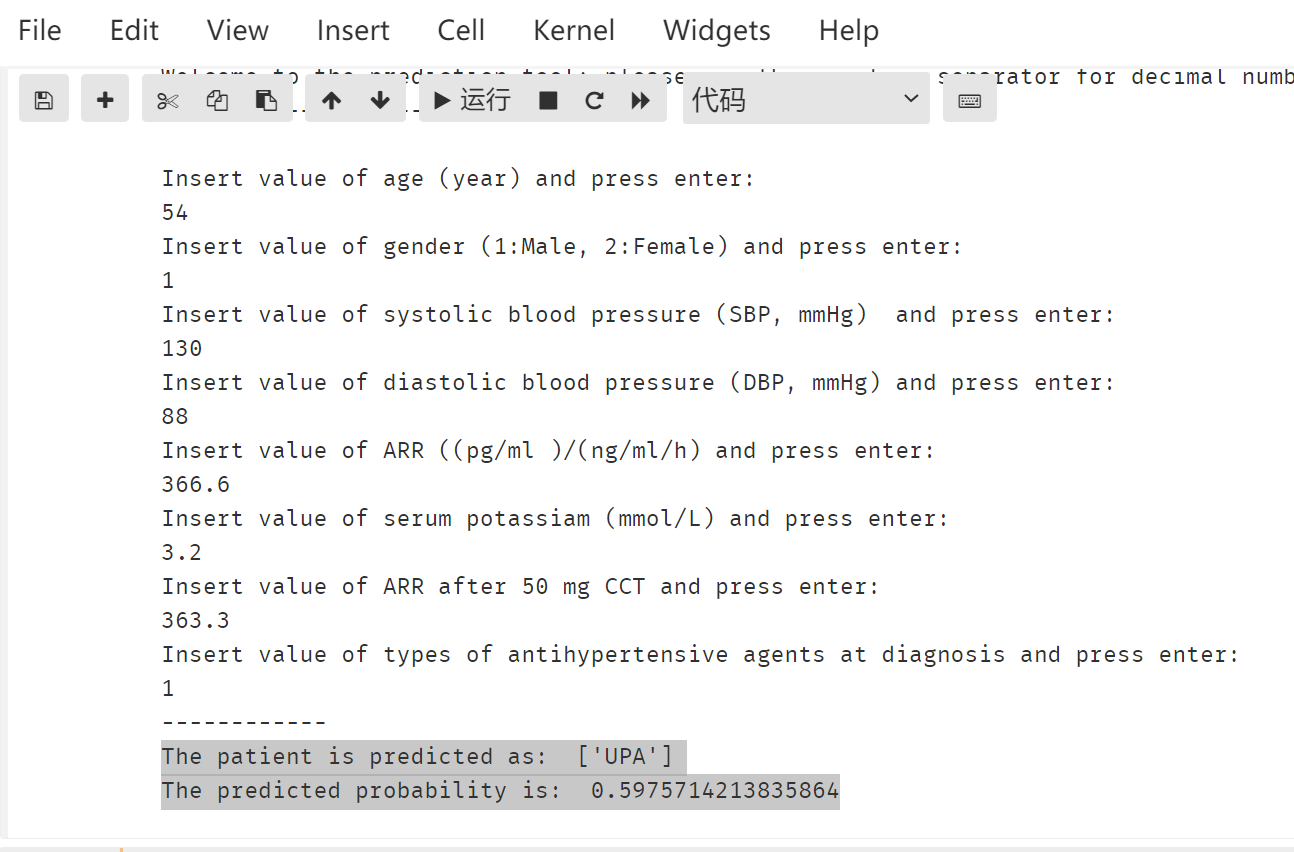

Supplement: Supplementary file 1 [file DataSheet_1.zip › Supplementary files/Supplementary file 2 Application process of online tool.docx]
