# Supplementary figures and images for: A new machine learning-based prediction model for subtype diagnosis in primary aldosteronism
Source: Front Endocrinol (Lausanne). 2022 Nov 23;13:1005934. doi: 10.3389/fendo.2022.1005934 (PMC9728523; doi:10.3389/fendo.2022.1005934)

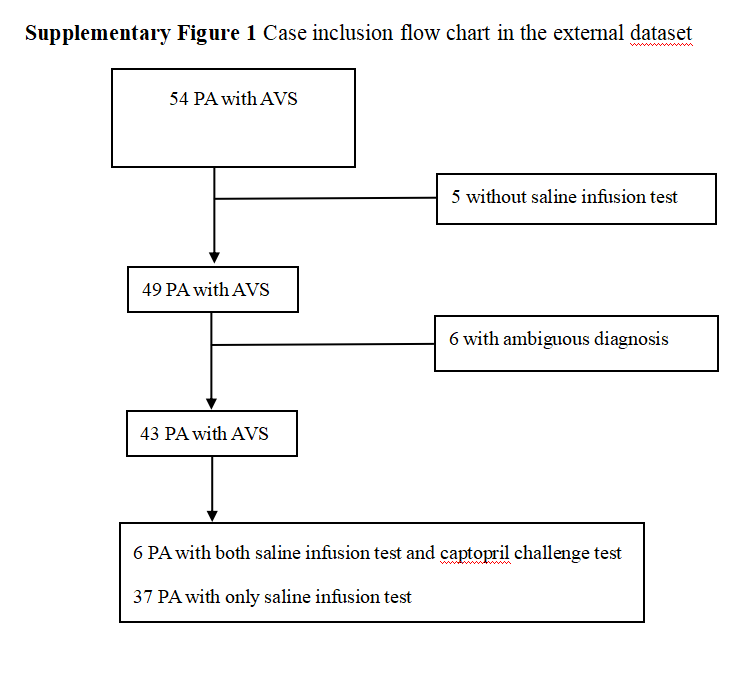

Supplement: Supplementary file 1 [file DataSheet_1.zip › Supplementary files/Supplementary Figure 1 .tif]

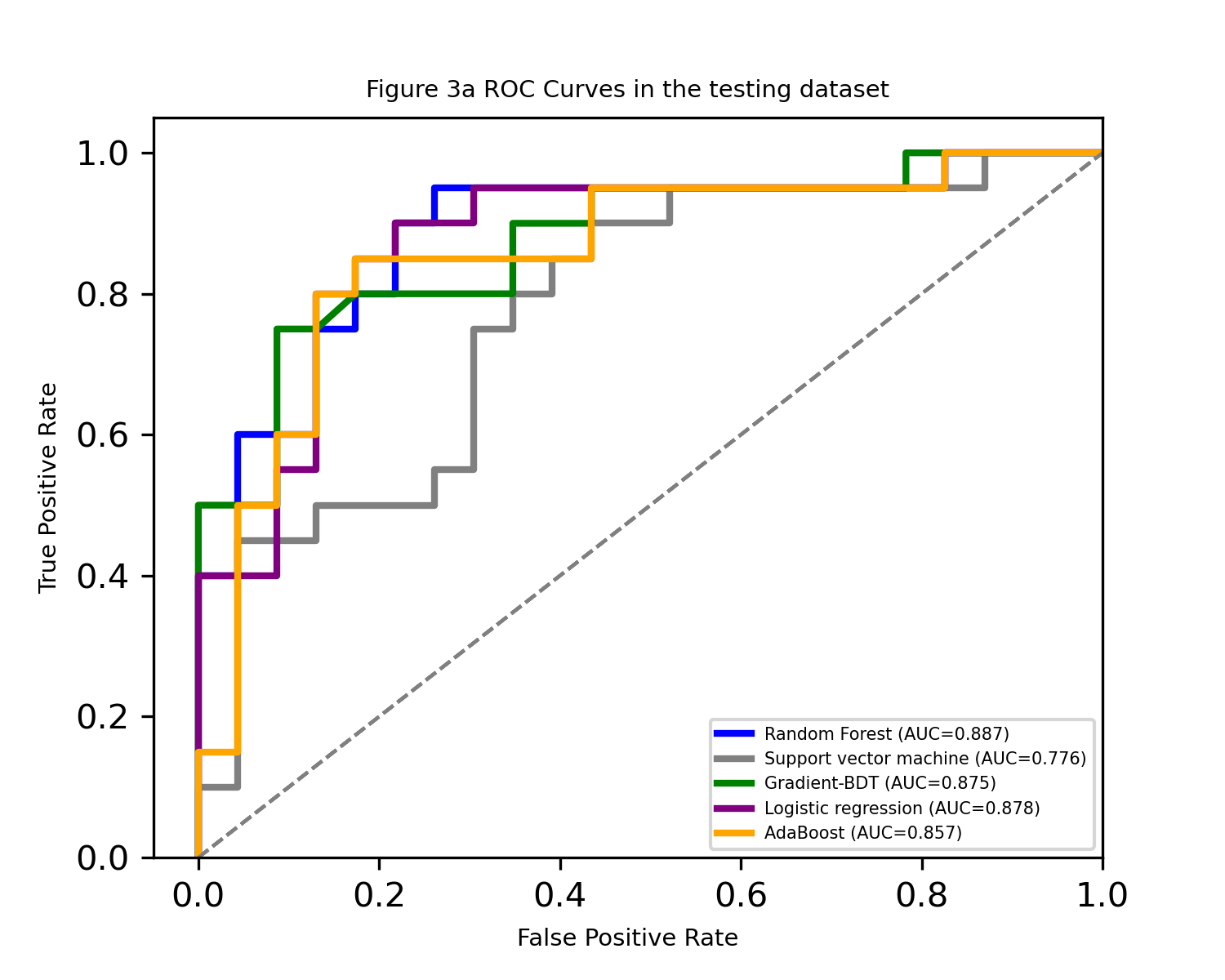

Supplement: Supplementary file 1 [file DataSheet_1.zip › Supplementary files/Supplementary Figure 2 ROC_external validation_all.png]

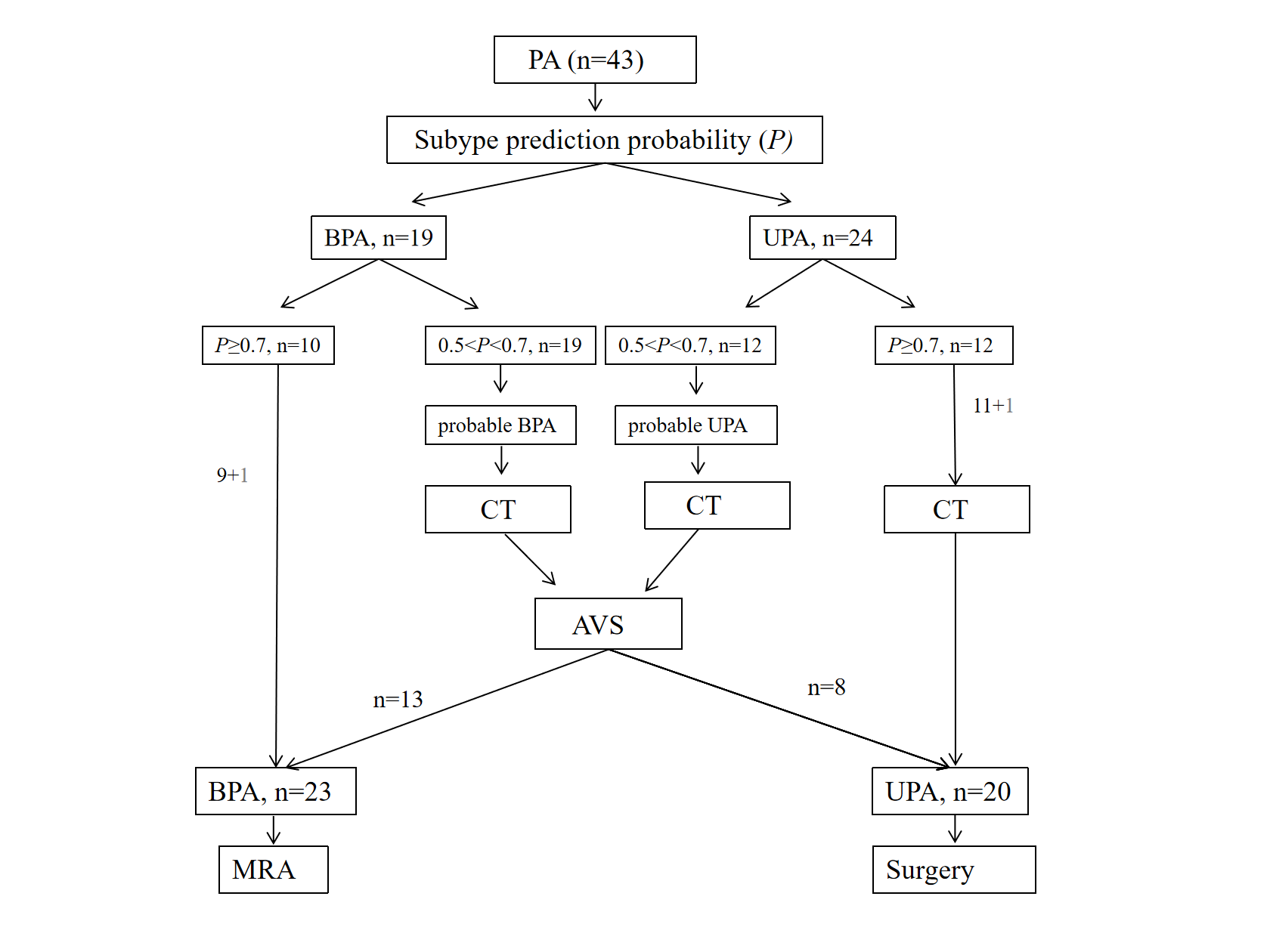

Supplement: Supplementary file 1 [file DataSheet_1.zip › Supplementary files/Supplementary Figure 3-Predicion model integrated with AVS.tif]

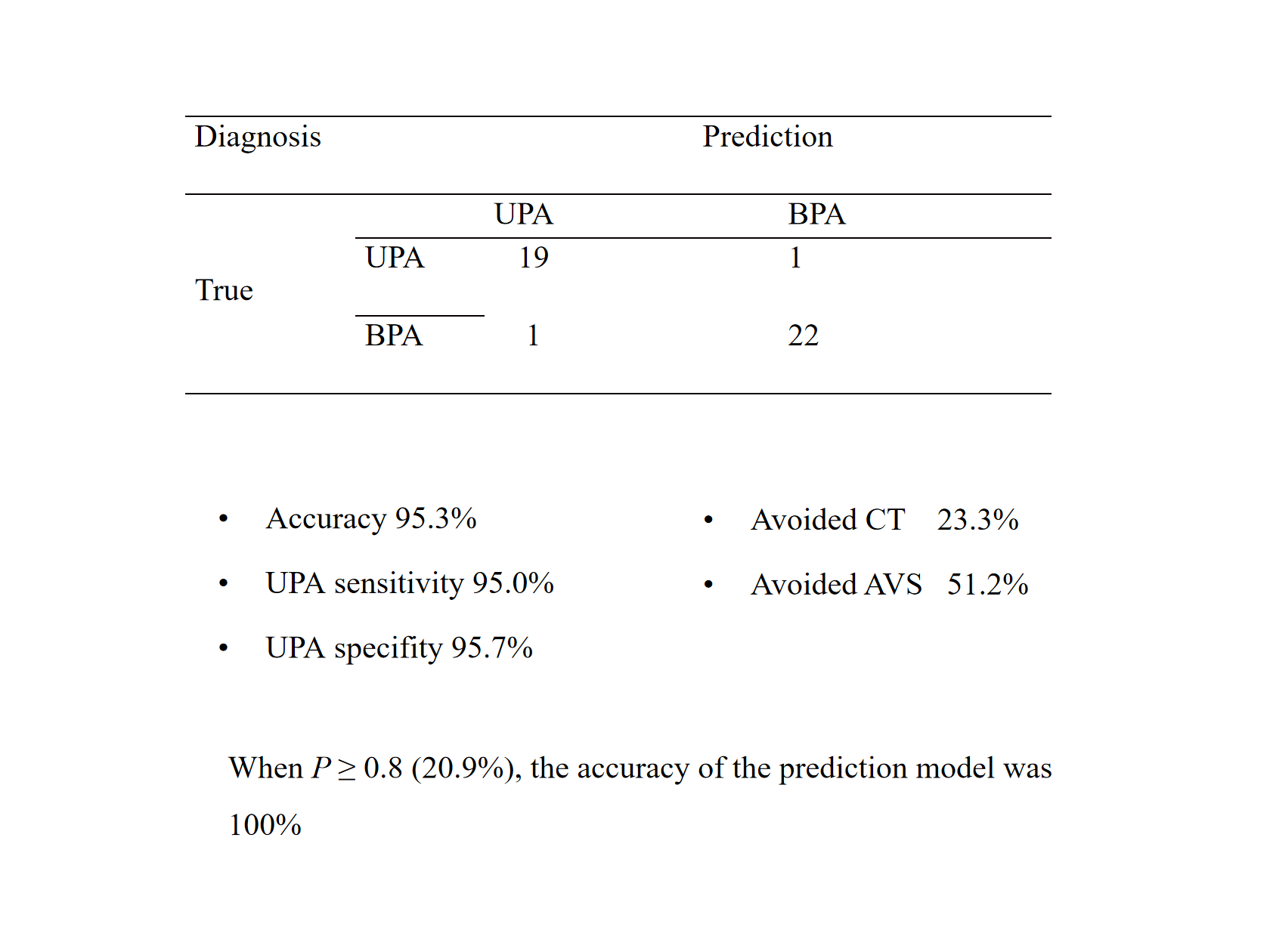

Supplement: Supplementary file 1 [file DataSheet_1.zip › Supplementary files/Supplementary Figure 4-Evaluation of integrated process.tif]

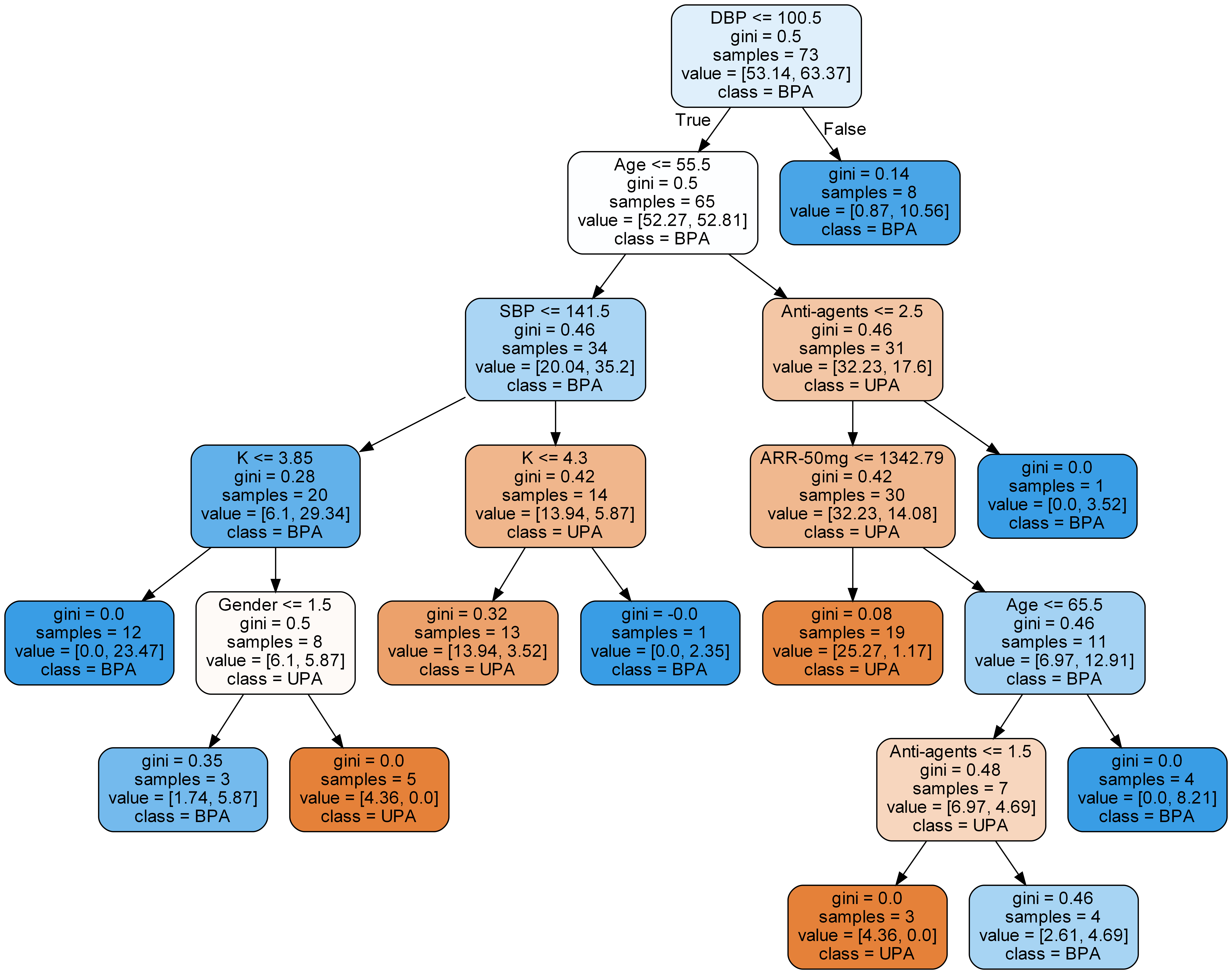

Supplement: Supplementary file 1 [file DataSheet_1.zip › Supplementary files/Supplementary folder 3 wiout SIT/c. one of the trees without SIT.png]

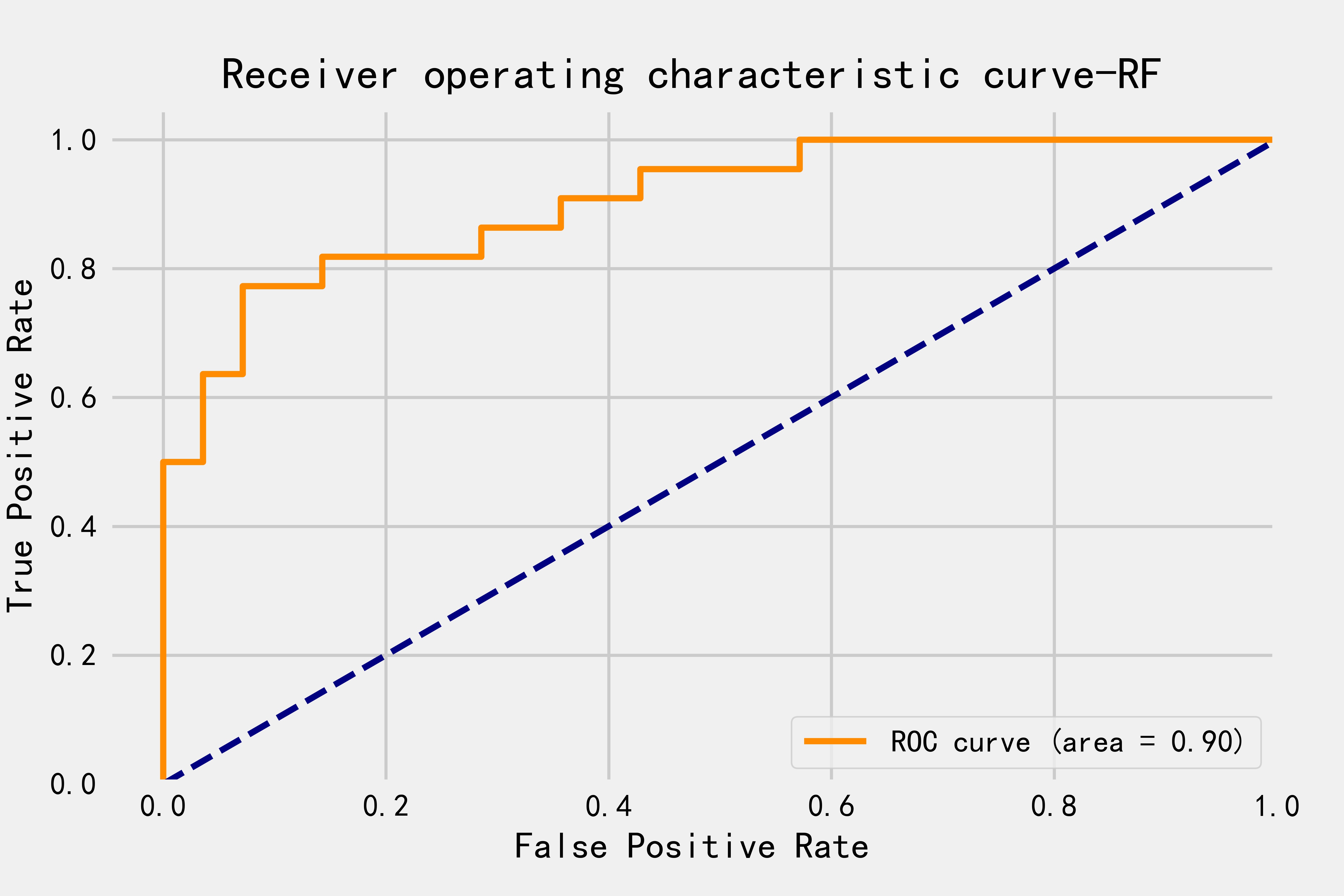

Supplement: Supplementary file 1 [file DataSheet_1.zip › Supplementary files/Supplementary folder 3 wiout SIT/d. ROC-RF without SIT.jpg]

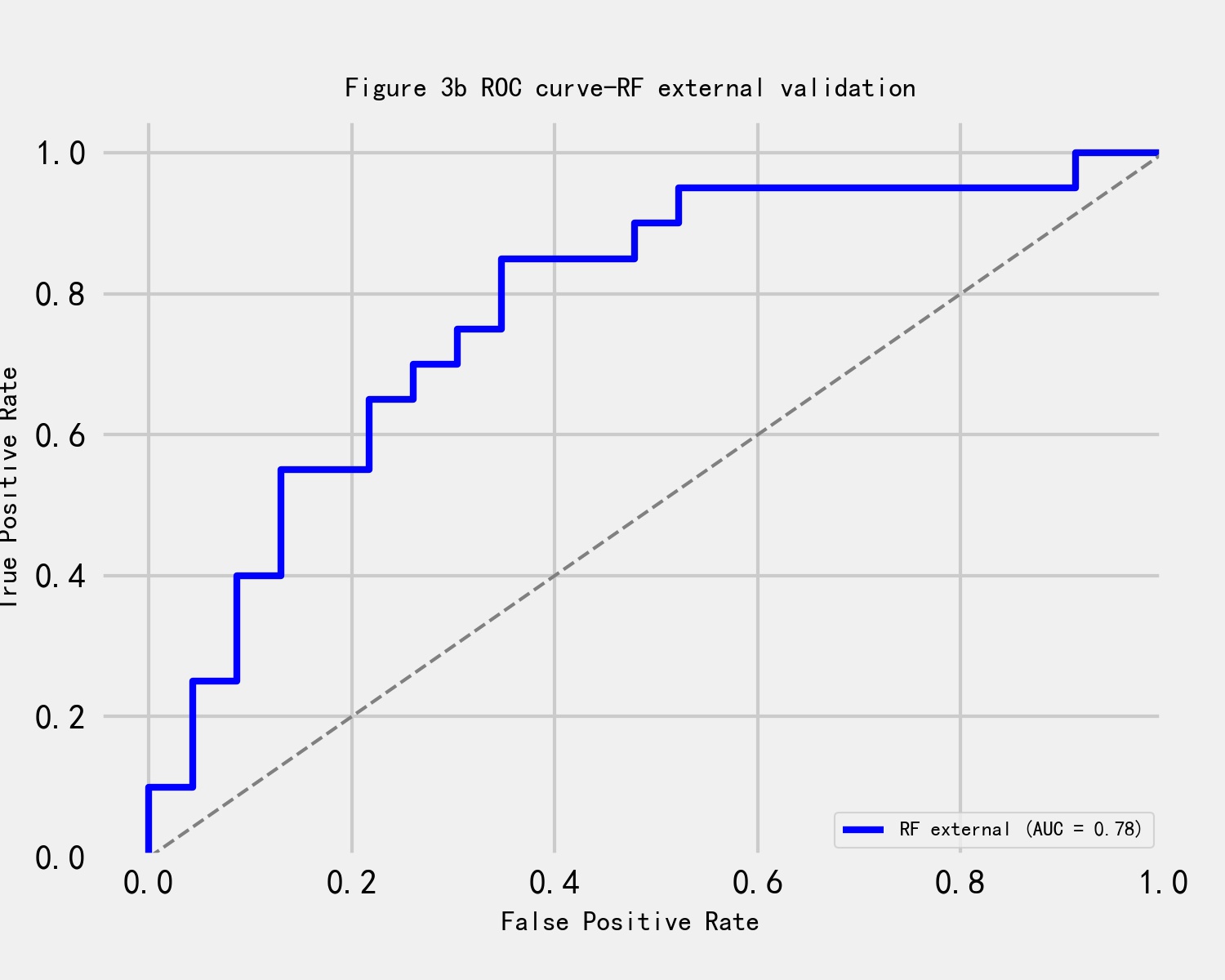

Supplement: Supplementary file 1 [file DataSheet_1.zip › Supplementary files/Supplementary folder 3 wiout SIT/e.ROC-RF external without SIT.jpg]
