# Supplementary material for: A new machine learning-based prediction model for subtype diagnosis in primary aldosteronism
Source: Front Endocrinol (Lausanne). 2022 Nov 23;13:1005934. doi: 10.3389/fendo.2022.1005934 (PMC9728523; doi:10.3389/fendo.2022.1005934)
Supplement: Supplementary file 1 [file DataSheet_1.zip › Supplementary files/Index of supplementary files.doc]

**Index of supplementary files**

1. **Supplementary Figure 1**: Case inclusion flow chart in the external dataset
2. **Supplementary Figure 2**: ROC curves of external validation with other classifiers
3. **Supplementary Figure 3**: The workflow of the developed model integrated with AVS to discriminate between UPA and BPA in the external dataset
4. **Supplementary Figure 4**: The performance of the workflow which integrated the developed model with AVS
5. **Supplementary Table 1:** Basic characteristics of primary aldosterone patients in the training dataset and validation dataset
6. **Supplementary Table 2:** Feature importance of Random Forest classifier
7. **Supplementary Table 3:** Available machine learning based prediction models for subtype diagnosis of PA
8. **Supplementary File 1**: Missing data details
9. **Supplementary File 2**: A demonstration of the application of the predictive model
10. **Supplementary File 3**: A demonstration of the application of the model without saline infusion test
11. **Supplementary Folder 1**: Datasets and process of the modelling before getting rid of the two parameters (urinary PAC and PRA after the furosemide standing test)
a. Modelling dataset with urinary PAC and PRA
b. External dataset with urinary PAC and PRA
c. Python code for the model using RF classifier with urinary PAC and PRA
12. **Supplementary Folder 2**: Python source codes for the development process
a. Python code for the model using RF classifier and external validation
b. Python code for the model using SVM classifier
c. Python code for the model using BDT classifier
d. Python code for the model using LR classifier
e. Python code for the model using Ada classifier
f. Python code for the ROC curves
g. Python code for ROC curve in the external validating dataset
h. Python code for the website application
i. Python code for the model using RF classifier without SIT
j. Python code for the website application without SIT
13. **Supplementary Folder 3**: Development and evaluation of prediction model for PA subtype diagnosis without SIT
a. Modelling dataset without SIT
b. External dataset without SIT
c. One of the prediction trees in the model using RF classifier without SIT
d. ROC curve in the testing dataset for the model using RF classifier without SIT
e. ROC curve in the external dataset for the model using RF classifier without SIT
f. Relative variable importance for the accuracy of predicting UPA using RF classifier without SIT
