# Supplementary material for: A new machine learning-based prediction model for subtype diagnosis in primary aldosteronism
Source: Front Endocrinol (Lausanne). 2022 Nov 23;13:1005934. doi: 10.3389/fendo.2022.1005934 (PMC9728523; doi:10.3389/fendo.2022.1005934)
Supplement: Supplementary file 1 [file DataSheet_1.zip › Supplementary files/Supplementary File 1- missing data description.doc]

**Missing data details**

(1) **Modeling dataset**

After enrollment, missing values were only present in 10 patients (6.1%) that lacked an aldosterone-to-renin ratio (ARR) after the 50 mg captopril challenge test (CCT) and 1 patient (0.6%) that lacked the number of types of antihypertensive agents at diagnosis; these missing values were replaced with the mean value.

(2) **External dataset**

There was no missing data.
