# Supplementary material for: A new machine learning-based prediction model for subtype diagnosis in primary aldosteronism
Source: Front Endocrinol (Lausanne). 2022 Nov 23;13:1005934. doi: 10.3389/fendo.2022.1005934 (PMC9728523; doi:10.3389/fendo.2022.1005934)
Supplement: Supplementary file 1 [file DataSheet_1.zip › Supplementary files/Supplementary file 3 PA group.doc]

**Appendix S1.**

**Primary Aldosteronism Collaborative Research Group of Xiangyang Central Hospital**

**Principal investigator:** Shaoyong Xu

**Consulting Members:**

*Center for Clinical Evidence-Based and Translational Medicine:* Bo Chen, Jingjing Zeng.

**Study Group Field Centers** (all field centers contributed equally):

*Department of Endocrinology*: Shaoyong Xu (Principal investigator), Shaomin Shi, Wan Xu, Mengyun Lei.

*Department of General Medicine*: Qing’an Li (Sub-investigator), Ming Yu, Luhong Li.

*Department of Cardiology:* Siyu Guan (Sub-investigator), Yong Ren, Hui Zhao.

*Department of Interventional Medicine:* Jingzhong Wang (Sub-investigator), Guangyuan Cheng, An Li, Jun Han, Xiaoqi Li.

*Department of Urinary Surgery:* Xuejun Zhang (Sub-investigator), Ji Zhou, Mingwei Xu.

*Research Assistant:* Anqi Wang, Ying Li, Wenlei Xu.
