# Supplementary material for: A new machine learning-based prediction model for subtype diagnosis in primary aldosteronism
Source: Front Endocrinol (Lausanne). 2022 Nov 23;13:1005934. doi: 10.3389/fendo.2022.1005934 (PMC9728523; doi:10.3389/fendo.2022.1005934)
Supplement: Supplementary file 1 [file DataSheet_1.zip › Supplementary files/Supplementary Table 1.doc]

Supplementary Table 1 Basic characteristics of primary aldosterone patients in the training dataset and validation dataset

|  | Modeling dataset(165) | | |
| --- | --- | --- | --- |
|  | Training dataset  (115) | Testing dataset  (50) | *P* |
| Age (year) | 54.0±11.0 | 54.2±10.5 | 0.924 |
| Gender(Male%) | 61(52.6%) | 18(36.7%) | 0.063 |
| SBP(mmHg) | 139±16.5 | 143±20.9 | 0.159 |
| DBP(mmHg) | 86±11.8 | 8786±11.14.4 | 0.676 |
| ARR | 1052±1545 | 1061±11421 | 0.975 |
| K(mmol/L) | 4.2±5.5 | 3.6±0.6 | 0.464 |
| ARR after 50 mg captopril loading | 1432±2562 | 1541±339 | 0.799 |
| PAC after SIT | 164.7±233.8 | 218.1±368.6 | 0.265 |
| PAC reduction after SiT (%) | 39.5±50.7 | 32.4±64.3 | 0.456 |
| Antihypertensive agents at diagnosis | 1.41±0.81 | 1.51±0.98 | 0.478 |

Abbrevations: SBP indicates systolic blood pressure; DBP, diastolic blood pressure; ARR, aldosterone to renin ratio; K, serum potassium level; PAC, plasma aldosterone concentration; SIT, saline infusion test.
