# Supplementary material for: A new machine learning-based prediction model for subtype diagnosis in primary aldosteronism
Source: Front Endocrinol (Lausanne). 2022 Nov 23;13:1005934. doi: 10.3389/fendo.2022.1005934 (PMC9728523; doi:10.3389/fendo.2022.1005934)
Supplement: Supplementary file 1 [file DataSheet_1.zip › Supplementary files/Supplementary Table 2.doc]

**Supplementary Table 2** Feature importance of Random Forest classifier

| Age | Gender | SBP | SBP | ARR | K | ARR-50mg | PAC-SIT | PAC reduction | Anti-agents |
| --- | --- | --- | --- | --- | --- | --- | --- | --- | --- |
| 0.039 | 0.007 | 0.045 | 0.054 | 0.219 | 0.088 | 0.183 | 0.271 | 0.088 | 0.006 |
