# Supplementary material for: A new machine learning-based prediction model for subtype diagnosis in primary aldosteronism
Source: Front Endocrinol (Lausanne). 2022 Nov 23;13:1005934. doi: 10.3389/fendo.2022.1005934 (PMC9728523; doi:10.3389/fendo.2022.1005934)
Supplement: Supplementary file 1 [file DataSheet_1.zip › Supplementary files/Supplementary Table 3.doc]

**Supplementary Table 3** Available machine learning based prediction models for subtype diagnosis of PA

| Study ID  (Country) | Predict target | Sample  Number | Number: Included features | important variables | Optimal Classifier | Predictive performance for UPA in testing dataset  (external validation) | | | | |
| --- | --- | --- | --- | --- | --- | --- | --- | --- | --- | --- |
|  |  |  |  |  |  | Accuracy | Sensitivity | Specificity | F1 score | AUC |
| 2021Buffolo^10^  (Italy) | UPA  (also PA) | M:377  (130/247)  E:129 | 6: Male, sex, SBP, antihypertensive treatment, BMI, lowest potassium and organ damage | serum potassium, organ damage, DDD | RF | 0.820  (0.753) | 0.821  (0.778) | 0.819  (0.750) |  | 0.905  (0.844) |
| 2021Kaneko^14^  (Japan) | UPA in general practice | M:229  (91/138)  E:120 | 21: Age, BMI, SBP, DBP, PAC, PRA, AST, ALT, Alb, UA, UN, eGFR, TC, HDL-C, LDL-C, TG, BS, Na, K, Cl, Ca | PAC, serum K, Na | RF | 0.957  (0.883) | 0.944  (0.690) | 0.964  (0.945) |  | 0.990  (0.872) |
| 2020Burrello^15^  (Italy) | UPA | M:215  (133/82)  E:118 | 6 : PAC at screening, after confirmatory testing, lowest K, presence of nodules, nodule diameter, and CT results | nodule diameter, lowest K, presence of nodules | RF | 0.870 (0.805) |  |  |  |  |
| 2020Eisenhofer^12^ (Germany) | UPA due to KCNJ5 variants  (also PA) | 273(139/134)  M: (90%)  E:10% | 7: MS–based plasma steroids: aldosterone, androstenedione, 18- hydroxycortisol, cortisone, 11-deoxycortisol, 18-oxocortisol, 11-deoxycorticosterone | aldosterone, 18-oxocortisol, and 18-hydroxycortisol | SVM |  | 0.850  (1.000) | 0.970  (0.980) | 0.801  (0.923) | 0.909 (0.991) |
| 2020Burrello^11^  (Italy) | UPA when  AVS is  unilaterally successful | M:158  (110/48)  E:- | 6: PAC at screening, after confirmatory testing, lowest K, normal ipsilateral CT imaging, normal contralateral CT imaging, contralateral A/C at AVS | contralateral A/C at AVS, normal ipsilateral CT imaging, lowest K | LDA | 0.846 | 0.847 | 0.842 |  |  |
| present  (China) | UPA bypass AVS | M:165  (71/94)  E:43 | 10: Age, gender, SBP, DBP, ARR, K, ARR after CCT, PAC after SLT, PAC reduction rate after SIT, antihypertensive agents at diagnosis | PAC after SIT, ARR, ARR after CCT and K | RF | 0.898  (0.814) | 0.857  (0.850) | 0.929  (0.783) | 0.878  (0.810) | 0.935  (0.883) |

Abbrevations: ID indicates identification, UPA, ulilateral primary aldosteronism; BPA, bilateral primary aldosteronism; PA, primary aldosteronism; M, sample number in the modeling dataset (number of UPA/number of BPA); E, sample number in the external dtaset; AUC, the area under the receiver operating characteristic curve; BMI, body mass index; SBP, systolic blood pressure; DBP, diastolic blood pressure; DDD, Defined Daily Dose(average maintenance dose per day for a drug used for its main indication in adults); RF, random forest; PAC, plasma aldosterone concentration; PRA, plasma renin activity; AST, aspartate aminotransferase; ALT, alanine aminotransferase; Alb, albumin; UA, uric acid ; UN, urea nitrogen; eGFR, estimated glomerular fltration rat; TC, total cholesterol; HDL-C, high density lipoprotein cholesterol; LDL-C, low density lipoprotein cholesterol; TG, triglyceride; BS, blood sugar; Na, serum sodium level; K, the lowest K level; Cl, serum chlorine level; Ca, serum calcium level; CT, computed tomography; MS, mass spectrometty; SVM, support vector machines; AVS, adrenal venous sampling; A/C, aldosterone/cortisone ratio; LDA, linear discriminant analysis; ARR, aldosterone to renin ratio; CCT, captopril challenge test; SIT, saline infusion test.
